# “Be sustainable”: EOSC‐Life recommendations for implementation of FAIR principles in life science data handling

**DOI:** 10.15252/embj.2023115008

**Published:** 2023-11-15

**Authors:** Romain David, Arina Rybina, Jean‐Marie Burel, Jean‐Karim Heriche, Pauline Audergon, Jan‐Willem Boiten, Frederik Coppens, Sara Crockett, Katrina Exter, Sven Fahrner, Maddalena Fratelli, Carole Goble, Philipp Gormanns, Tobias Grantner, Björn Grüning, Kim Tamara Gurwitz, John M Hancock, Henriette Harmse, Petr Holub, Nick Juty, Geoffrey Karnbach, Emma Karoune, Antje Keppler, Jessica Klemeier, Carla Lancelotti, Jean‐Luc Legras, Allyson L Lister, Dario Livio Longo, Rebecca Ludwig, Bénédicte Madon, Marzia Massimi, Vera Matser, Rafaele Matteoni, Michaela Th Mayrhofer, Christian Ohmann, Maria Panagiotopoulou, Helen Parkinson, Isabelle Perseil, Claudia Pfander, Roland Pieruschka, Michael Raess, Andreas Rauber, Audrey S Richard, Paolo Romano, Antonio Rosato, Alex Sánchez‐Pla, Susanna‐Assunta Sansone, Ugis Sarkans, Beatriz Serrano‐Solano, Jing Tang, Ziaurrehman Tanoli, Jonathan Tedds, Harald Wagener, Martin Weise, Hans V Westerhoff, Rudolf Wittner, Jonathan Ewbank, Niklas Blomberg, Philip Gribbon

**Keywords:** Computational Biology, Methods & Resources, Science Policy & Publishing

## Abstract

The main goals and challenges for the life science communities in the Open Science framework are to increase reuse and sustainability of data resources, software tools, and workflows, especially in large‐scale data‐driven research and computational analyses. Here, we present key findings, procedures, effective measures and recommendations for generating and establishing sustainable life science resources based on the collaborative, cross‐disciplinary work done within the EOSC‐Life (European Open Science Cloud for Life Sciences) consortium. Bringing together 13 European life science research infrastructures, it has laid the foundation for an open, digital space to support biological and medical research. Using lessons learned from 27 selected projects, we describe the organisational, technical, financial and legal/ethical challenges that represent the main barriers to sustainability in the life sciences. We show how EOSC‐Life provides a model for sustainable data management according to FAIR (findability, accessibility, interoperability, and reusability) principles, including solutions for sensitive‐ and industry‐related resources, by means of cross‐disciplinary training and best practices sharing. Finally, we illustrate how data harmonisation and collaborative work facilitate interoperability of tools, data, solutions and lead to a better understanding of concepts, semantics and functionalities in the life sciences.

## Graphical abstract

Creating a space for open collaborative research, EOSC‐Life, suggests best practices for sustainable data management for enhanced usage of life science resources.

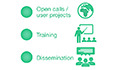

## Introduction

Life Science (LS) communities cover multiple scientific domains and carry out a diversity of research, from basic biological studies to applied epidemiological and environmental investigations. This breadth is evidenced by the key role played by LS communities during the COVID‐19 pandemic, ranging from fundamental studies of the SARS‐CoV‐2 virus, the discovery of new therapies and the development of novel vaccines, to establishing and validating methods for contact tracing and wastewater surveillance. The COVID‐19 pandemic demonstrated that **LS communities can provide high‐quality, reliable data** for reuse by the wider scientific community (see recommendations from Research Data Alliance (RDA) COVID‐19 Working Group, 2020). LS communities around the world have, however, voiced the need to improve the sharing of, access to and, ultimately, reuse of data resources in a FAIR (Findable, Accessible, Interoperable and Reusable; Wilkinson *et al*, 2016) manner. Increased data sharing and accessibility would: (i) enhance the value of results generated within a specific domain and expand their utility by integrating data from related communities; (ii) provide a vital data substrate that **supports non‐hypothesis‐driven** scientific discovery and advances through the application of machine‐based methods and (iii) inform decision making by policymakers (e.g. funders, politicians) and industrial partners.

To facilitate research, in addition to FAIR data, there is a need for FAIR research software (Lamprecht *et al*, 2020; Barker *et al*, 2022; Chue Hong *et al*, 2022). Software is used in almost all areas of science and must be sustainable to guarantee the reproducibility and reusability of the results, data and analyses it generates. This requires long‐lived, executable software, which in turn necessitates funds, typically only available through fixed‐term project calls that value novelty above utility. This problem has, however, been recognised by funders (Strasser *et al*, 2022) and has led to the Amsterdam Declaration on Funding Research Software Sustainability that aims to change comprehensively the way funders deal with research software (https://zenodo.org/records/8325436).

EOSC‐Life is a European project funded under Horizon 2020. It brings together 13 LS Research Infrastructures (RIs) from the European Strategy Forum on Research Infrastructures (ESFRI) to create an open, digital and collaborative space for biological and medical research. The project publishes FAIR data and a catalogue of services provided by participating RIs that enable the management, storage and reuse of data in the European Open Science Cloud (EOSC; preprint: Appleton *et al*, 2020). The project is framed by a data management plan describing the minimum requirements necessary to initiate research data sharing (Blomberg *et al*, 2020 in Appendix 1). The project participants, however, have recognised that merely using this tool will not ensure the long‐term and large‐scale use of scientific data. EOSC‐Life has identified organisational, technical, financial and legal/ethical challenges that represent the main barriers to effective sustainability (definitions in Box 1).

Box 1 **Definitions for sustainability types addressed in this paper.**

- Organisational sustainability: (i) organisations create and support data, as well as services to maintain that support and (ii) organisations continue to support their members who are making the efforts to produce FAIR data, services, software, etc.
- Technical sustainability: metadata, data, software, workflows, AAI (Authentication and Authorization Infrastructure), etc., need to be future proof and flexible enough to continue to be used even as technologies evolve.
- Financial sustainability: the financial resources to provide for the human resources and the storage and operational space (cloud infrastructure, supercomputing, computer storage with IT support).
- Sustainability through continued re‐use: data, tools and software continue to be used because they can be found, accessed, operated on and re‐used, and so do not grow stale. Also, building on existing solutions, i.e. established user‐bases, as opposed to redundant innovation.
- Sustainability through training: tools and software can continue to be used because they are actively disseminated and explained through training, and where the training serves also to identify the new needs of old and new users in emerging communities and emerging scientific domains.

The **organisational challenges** are associated with the non‐aligned impact and reward mechanisms operating within academic organisations. Here, pursuing novelty and following new trends are more likely to result in positive funding or tenure decisions compared to investments in community‐oriented collaborative initiatives addressing long‐term structural issues. The **technical challenges** are related to the need to promote the effective, widespread awareness and implementation of FAIR components, including the need to address sustainability dependencies arising from operating systems, versioning, identifiers, metadata schemata, vocabularies and provenance. These components are often inconsistently applied, hindering interoperability and reducing opportunities for reuse of both data and software. The **financial challenges** arise from the proliferation of services and LS data repositories (between 2020 and 2023, for example, the *Nucleic Acids Research* (https://www.oxfordjournals.org/nar/database/c/) Database issue reported 267 new ones) and the increasing size and complexity of datasets and services. Consequently, the resources available to perform basic operations, including curation, storage, computing and access, may be insufficient. **Ethical and Legal challenges** include the need to respect intellectual property rights and comply with the GDPR (General Data Protection Regulation; https://gdpr‐info.eu/) in a fragmented international legal landscape. How these challenges are to be overcome when a project ends is rarely addressed, leading to a reluctance to reuse resources once projects' primary objectives have been met.

In this paper, we describe resources and services, as well as the associated training and knowledge exchange, which have been supported and/or created within the EOSC‐Life project. We emphasise sustainability strategies formulated to ensure long‐term access to, and reuse of, these resources and services in a world where open‐science policies are being developed and where data and software are both shared and widely accessible. As EOSC‐Life is part of the wider EOSC ecosystem, we highlight special considerations and twelve key recommendations (**[R1–R12]**) for resource sustainability. These reflect the scientific diversity of EOSC‐Life's constituent communities, as well as external factors, such as the need to align with regulatory requirements related to personal health data.

The radical collaboration framework (McGovern, 2018a; Pickering *et al*, 2021) was used to assess the sustainability of EOSC‐Life outputs. We subsequently operationalised the methodology while developing this manuscript (Appendix Supplementary Information S1).

## Sustainability essentials, tools and challenges

### PART‐1: community components: “humans and data”

#### EOSC‐Life within the wider EOSC landscape

**Figure 1:**
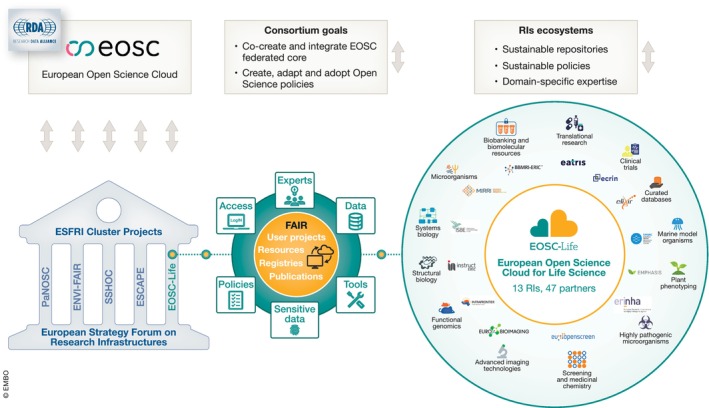
Position and organisation of EOSC‐Life in the EOSC and LS RIs ecosystems EOSC‐Life is one of five ESFRI Science Cluster projects (the other four clusters are PaNOSC, ENVRI‐FAIR, ESCAPE, SSHOC). EOSC brings together 13 LS RIs and domains experts to populate EOSC with LSs FAIR data, tools, resources, harmonised solutions, policies and guidelines and concrete user projects altogether facilitating interoperability in the EOSC (respective interactions and impact are illustrated by arrows).

The **EOSC** (Box 2; Fig 1) is a joint initiative from the European Commission (EC), its Member States, Associate Countries and stakeholders from European research communities. It aims to **federate access to research services and data** across scientific disciplines and international borders, under a common governance structure. The EOSC‐Life project exists within the wider EOSC ecosystem, creating interdependent sustainability approaches. These commonalities are explored by the EOSC Financial Sustainability, FAIR and Architecture Task Forces (https://eosc.eu/eosc‐task‐forces) and encourage the use of best practices in EOSC scientific clusters.

Box 2 **EOSC and connected communities.**

The ambition of the EOSC is to develop an “open multi‐disciplinary environment where researchers can publish, find and re‐use data, tools and services, thus enabling them to conduct their work better”. It builds on existing infrastructure and services in a federated “system of systems” approach. In order to link EOSC with efforts of the European Strategy Forum on Research Infrastructures (ESFRI) and other key European RIs, five Science Cluster projects were launched in 2019 and are structuring a large part of the EOSC research landscape (Lamanna *et al*, 2021 in Appendix 1):

- ENVRI‐FAIR (https://envri.eu/home-envri-fair/; environmental sciences)
- EOSC‐Life (https://www.eosc-life.eu/; life sciences)
- ESCAPE (https://projectescape.eu/; astronomy, astroparticle and particle physics)
- PaNOSC (https://www.panosc.eu/; photon and neutron sciences)
- SSHOC (https://sshopencloud.eu/; social sciences and humanities)

The science cluster projects have built on long‐standing interactions between the RIs in the different scientific domains and aim to improve researchers' access to data, tools and resources, as well as FAIR data management practices. Each science cluster project addresses domain‐specific requirements for linking their data resources to EOSC, but all of them also consider intra‐domain interoperability and alignment.

**EOSC‐Life facilitates and harmonises** the discovery, access and analysis of data for European and non‐European researchers across disciplines. It also provides ways to visualise data creatively, in models simulating reality. To achieve this, the EOSC‐Life consortium set out to:

- Publish data resources and associated metadata from LS RIs.
- Work with scientists who develop computational methods and packageable analysis tools and connect the latter to data.
- Develop guidelines and tools to be able to exploit sensitive data from human research participants in a secure manner.

The **EOSC‐Life** consortium has worked to shape and strengthen synergistic partnerships with RI users and external communities through **open calls for user projects** (https://www.eosc‐life.eu/calls/; Haley *et al*, 2020; Rybina *et al*, 2023 in Appendix 1), so as to:

- Create RI data resources, to support the work of individual facilities, or multidisciplinary consortia, to make data FAIR in the cloud.
- Partner with RIs to promote training and staff development.
- Encourage research communities to engage with EOSC‐Life to develop open services **[R11]**.
- Foster and support collaboration between academia and industry.
- Deal with sensitive data.

Domain experts in cloud computing, computational workflows, FAIR data, ethics and data management were assigned to dedicated project teams, supporting the implementation of projects by providing multidisciplinary expertise (Gribbon *et al*, 2020 in Appendix 1) **[R1]**. Through open calls, and working with existing RI‐managed data resources, EOSC‐Life created practical solutions that met the real needs of life scientists. Examples of outcomes, developed or enhanced during EOSC‐Life, are listed in the EOSC Association and the RDA Key Exploitable Results report (Nardello *et al*, 2022 in Appendix 1; Table 1 **) [R2]**.

**Table 1:** Key exploitable results from EOSC‐Life used by communities (Life Sciences Data Resources & Tools, harmonisation of Access & Policies—in EOSC).

Result	URL	Description
(i) LS Data in EOSC: FAIR LS data resources for cloud use (organisational sustainability, technical sustainability, sustainable access to data, sustainable data governance)		
COVID‐19 Portal	https://doi.org/10.25504/FAIRsharing.f3b7a9	Resources focusing on COVID‐19‐related data
Common Provenance Model	https://fairsharing.org/4610	Standard based on W3C PROV, which allows publishing, finding and interlinking provenance information in distributed environments
Clinical Research Metadata Repository	https://fairsharing.org/3067	Portal allows users to search for studies using a variety of criteria and identify the data objects associated with them
Ontology Lookup Service (OLS)	https://doi.org/10.25504/FAIRsharing.Mkl9RR	Repository for biomedical ontologies that aims to provide a single point of access to the latest ontology versions
Ontology Cross Reference Service (OxO)	https://doi.org/10.25504/FAIRsharing.0c6fea	Database of ontology cross‐references (or xrefs)
Sensitive data toolbox	https://fairsharing.org/3577	Open, digital and collaborative space for biological and medical research, across LS RIs
(ii) LS Toolkits in EOSC: eco‐system of innovative LS tools and key services/standards in EOSC (sustainability through re‐use, technical sustainability)		
FAIRsharing	https://doi.org/10.25504/FAIRsharing.2abjs5	Resource on (meta)data standards, inter‐related to databases and data policies
Workflowhub	https://doi.org/10.25504/FAIRsharing.07cf72	Registry for publishing scientific computational workflows
Schema.org	https://doi.org/10.25504/FAIRsharing.hzdzq8	Community activity handling schemas for structured data
Bioschemas.org	https://fairsharing.org/3517	LS branch of Schema.org aims to improve data interoperability in LS
RO‐Crate	https://doi.org/10.25504/FAIRsharing.wUoZKE	Community effort focusing on packaging research data with metadata, complementing richer metadata standards
FAIR Cookbook	https://faircookbook.elixir‐europe.org	Recipes for the FAIRification journey
Galaxy	https://galaxyproject.org/eu/	Open‐Source project for FAIR data analysis
Scipion (workflows)	https://doi.org/10.25504/FAIRsharing.EsY1WF	Cryo‐EM image processing framework
BAND	https://band.embl.de	Virtual Desktop for bioimage analysis in the cloud
RDMKit	https://rdmkit.elixir‐europe.org/	Best practices and guidelines to help you make your data FAIR
(iii) Harmonisation of Access and Policies across the LS (RIs)		
LS Login	https://lifescience‐ri.eu/ls‐login.html	Common Authentication and Authorisation Infrastructure (AAI**)** system for LS RIs and communities
ARIA	https://aria.services/	User access management system (AMS)
Open Calls Procedures Guidelines	https://zenodo.org/record/4048442	Guidelines for organising topic‐specific Open Calls (within EOSC framework)
Open Calls Recommendations	https://zenodo.org/record/8263074	Procedures and recommendations for integrating Open Calls and user projects into EOSC framework

#### Sustainability and governance

Governance concerns the responsibilities for managing research data and tools over the long term, in compliance with applicable regulations, as well as the allocation of suitable resources for data availability. **Governance structures** that engage research communities to promote best practices consistently are essential to ensure **long‐term accessibility** to research outputs **[R8]**. Here, research‐funding agencies play essential roles in catalysing best practices (Jahn *et al*, 2023 in Appendix 1). Many of them now require all data to be shared or deposited in specific repositories that apply FAIR and TRUST (Transparency, Responsibility, User‐Focus, Sustainability, Technology—Lin *et al*, 2020) principles. Best practices resulting from consultation with relevant user communities in governing data access, and resource sharing, should be an explicit part of operational management and risk mitigation (including cybersecurity) of research organisations, and of guidelines for infrastructures managing long‐term sustainable access to data. These aspects have been highlighted in several high‐level reports by, e.g. the Organisation for Economic Co‐operation and Development (https://legalinstruments.oecd.org/en/instruments/OECD‐LEGAL‐0463), G7, the Group of Senior Officials on global RIs (http://www.gsogri.org/) and the EC via ESFRI (https://www.esfri.eu/sites/default/files/u4/ESFRI_SCRIPTA_VOL2_web.pdf).

In this context, the core goal of EOSC‐Life is to facilitate data interoperability: to allow the interoperation and analysis of data across disciplines, whilst conforming to necessary governance and consent procedures. Data and metadata resources gain **added value** when they are **curated** by experts **[R1]**, well **annotated, processed** in sophisticated ways, and **integrated** into or linked to other datasets. For example, the value of sequencing data is enhanced when they are linked to the corresponding organism‐level functional data, allowing the connection to be made between genotype and phenotype e.g. for crop performance or human diseases. Such an approach is illustrated by the National Human Genome Research Institute—European Bioinformatics Institute catalogue of human genome‐wide association studies (https://www.ebi.ac.uk/gwas/; Sollis *et al*, 2023). To facilitate such data integration over time, and therefore promote sustainability through re‐use; however, interoperability between more disparate data types needs to be developed. Adequate data management is a key component of this interoperability. Minimising information loss and creating an auditable trail ensures data can be trusted. As interoperability also relies on the granting of permission to access and re‐use available data, it is essential that research outputs are accompanied by clear licencing information, and for the licences used to be as permissive as possible **[R11]**. In addition, the procedures and standards used to achieve added value must be clearly documented and made transparent to stakeholders, as was reported, for example, by the plant science community for the “MIAPPE” (https://www.miappe.org/; Minimum Information About a Plant Phenotyping Experiment) standard (Papoutsoglou *et al*, 2020) **[R2]**. As regulatory and ethical requirements have to be met, harmonising and simplifying data‐related legal frameworks across EOSC would also reduce frictions in data access and re‐use **[R9]**.

#### Sustainability at multiple scales

Smaller teams and projects from specialist communities, such as those integrated through EOSC‐Life Open Calls, often lack access to expertise in FAIR data management, in setting up sustainable hosting resources, or in implementing interoperability using available standards. This means newly established **resources risk being abandoned** after projects are completed. To address this, the EOSC‐Life open call process was structured to demonstrate first the utility of its support model for sustainable FAIR data management **[R3]** and then to explore more complex needs, including those of sensitive‐ and industry‐related data resources. Project applicants were asked, as part of the project design and implementation plan, to identify outcomes that would need to be sustained, and relevant domain experts, and then provided support and guidance **[R1]**. The sustainability of outcomes was analysed at the end of the project, in an iterative lessons‐learnt process, using “World‐Cafe (https://theworldcafe.com/key‐concepts‐resources/world‐cafe‐method/)” methodology. Supported by EOSC‐Life publications **[R2]** and training materials, many user projects contributed to improving the quality, functionality and scope of existing resources and created new resources, such as data repositories and standards. Significant impacts are described in Appendix Supplementary Information S2. Overall, this process highlighted the benefit of increasing awareness of existing sustainability solutions, and of training as a path to promote sustainability in small projects. In a fragmented environment of highly distributed data sources, both sustainability and data interoperability can be greatly enhanced by data integration into data warehouses. EOSC‐Life supported the initiation of large, centralised platforms, showing how these could be quickly built to meet urgent community and societal needs **[R6]** e.g. the COVID‐19 Data Portal (https://www.covid19dataportal.org/; April 2020) enabling researchers to upload, access and analyse COVID‐19‐related reference data, and specialist datasets, as part of the wider European COVID‐19 Data Platform (Appendix Supplementary Information S3 and **[R6]**).

#### Sustaining FAIR by connecting communities

EOSC‐Life provides a framework for 13 LS RIs at different stages of organisational development to establish partnerships and ensure service interoperability. These RIs represent research communities with different levels of adoption of Open‐Science and FAIR practices. Certain RIs have produced white papers that map their possible paths to sustainability, e.g. ELIXIR (https://elixir‐europe.org/, https://f1000research.com/documents/8‐1642). The exchange of knowledge among RIs should evolve into common organisational sustainability strategies **[R8]**. For instance, Memoranda of Understanding (e.g. between ELIXIR and BBMRI (https://www.bbmri‐eric.eu/), or EU‐OPENSCREEN (https://www.eu‐openscreen.eu/), EURO‐BIOIMAGING (https://www.eurobioimaging.eu/) and INSTRUCT (https://instruct‐eric.org/); https://www.eu‐openscreen.eu/newsroom/eu‐openscreen‐news/ansicht/eu‐openscreen‐has‐signed‐a‐memorandum‐of‐understanding‐mou‐with‐eurobioimaging‐and‐instruct‐eric.html) have been established to formalise cross‐RI partnerships.

**Figure 2:**
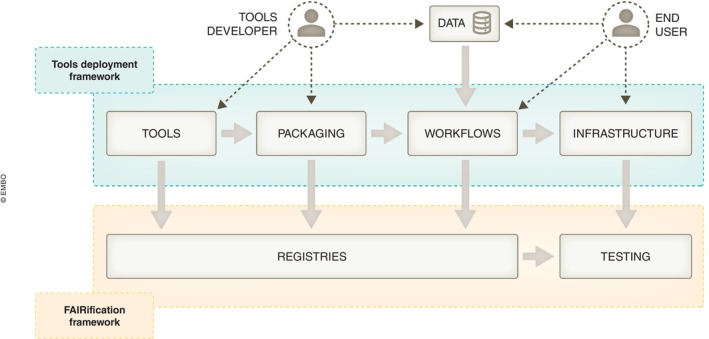
EOSC‐Life operational framework, extending from the end user to the EOSC ecosystem Dashed lines indicate use of and contributions to the framework, while thick arrows indicate process flow within the framework.

RI‐associated communities have also learnt about different Open‐Science and FAIR practices, through EOSC‐Life activities. Initial work highlighted the steps required to ensure a common use of open formats and ontologies by RIs and the need to develop Application Programming Interfaces (APIs) for data exchange. As a pilot for interoperable resources, graph databases and knowledge visualisations built around aligned metadata standards were developed for COVID‐19 and Monkeypox (Karki *et al*, 2023) **[R5]**. Technical sustainability was addressed by including outcomes in enduring resources such as the FAIR Cookbook (https://faircookbook.elixir‐europe.org; Appendix Supplementary Information S4) and RDMKit (https://rdmkit.elixir‐europe.org/; ELIXIR, 2021 in Appendix 1), which integrate findings from multiple academic and industrial projects, provide recipes for FAIRification and tools for Research Data Management (RDM), respectively. These community projects support Open‐Science and ensure that access to materials is sustained beyond a single project **[R2]**. EOSC‐Life also reached out across communities to promote an inclusive framework **[R11]** (Fig 2) supporting the interoperability and portability of software tools and computational workflows from different domains, through the use of a common set of technologies, such as software containerisation. Gaps in the FAIRification of computational workflows were addressed by creating the **WorkflowHub** (https://workflowhub.eu), a registry for **workflows in a highly federated ecosystem** of different workflow managers and community‐owned repositories (Goble *et al*, 2023 in Appendix 1). By using Research Object (RO)‐Crates, lightweight packages of research data with their metadata, and established web approaches (schema.org, JSON‐LD, Common Workflow Language, and GA4GH APIs) **[R9]**, workflows can be exchanged between services for their execution and testing (see LifeMonitor (https://app.lifemonitor.eu/) in Appendix Supplementary Information S6). They can also be exported to long‐term archives and described so that they can be reimplemented or executed in alternate systems **[R5]**.

### PART‐2: technical components developed to support sustainability

#### EOSC‐Life sustainable Open‐Science toolkit for FAIR RDM services

FAIR interoperability services ensure that (meta)data use a formal, accessible, shared and broadly applicable language to represent knowledge, typically relying on vocabularies or ontologies that follow FAIR principles and make qualified references to other (meta)data. They are needed for the re‐use of data and tools, but also to address the complexity of the biomedical domain, ranging from the diversity of samples and data‐generating technologies to variable granularity of datasets. **Ontologies should be accessible**, e.g. via the Ontology Lookup Service (https://www.ebi.ac.uk/ols4; OLS; see Appendix Supplementary Information S5), and the annotations must have web‐resolvable Persistent and unique IDentifiers (PIDs), as recommended by the EOSC Association Task Force on PID Policy and Implementation (https://www.eosc.eu/advisory‐groups/pid‐policy‐implementation) **[R5]**. Mappings between ontologies, vocabulary schema or coding standards are important to ensure the quality and sustainability of the metadata. EOSC‐Life implemented interoperability services such as the Ontology Cross Reference Service (https://www.ebi.ac.uk/spot/oxo) in the form of a suite of semantic services, deployed using cloud principles and technologies (Appendix Supplementary Information S5) **[R9]**. Sustainability has been achieved by extending and evolving pre‐existing services, as well as by developing new services to answer multiple user needs. Cloud deployment has become accessible and affordable to all, and some datasets/data resources are being deployed in industrial or secure environments, highlighting the importance of **making interoperability services portable** to increase their sustainability.

#### Sustainability of sharing and shared (meta)data

Data and software must be archived in **online portals** along with rich and semantically annotated **metadata** to be **findable and usable** over the long term. Sustainability through re‐use requires that the data contain, or be intimately linked to, their own metadata to ensure that the data are useful and understandable, even after they have been downloaded from the portal **[R5]**. Open specifications of data formats, semantics, provenance and machine‐actionable data content are the cornerstone of sustainable data interoperability, by making data accessible to all users and services, without tying data exchange and processing to specific hardware, software or groups. Open specifications offer guarantees against obsolescence as they can evolve with community needs, and new implementations can be made available as technologies evolve **[R11]**. EOSC‐Life contributed to identify EOSC Semantic Interoperability challenges in the framework of EOSC Task Forces (David *et al*, 2023a) and to increase domain coverage, for example, with the Recommended Metadata for Biological Images (Sarkans *et al*, 2021), or for microbial resources, the development of a unique identification system based on Digital Object Identifier (DOI; Romano *et al*, 2022 in Appendix 1). This identification system enables the users to retrieve microbial metadata (including ecological and legal data) from Data MIRRI dataportal and potentially connect to other data housed in other RI **[R5]**.

#### Curation of FAIR data resources and collections

The constant curation of data resources ensures the quality, accuracy and depth of data, improves their reuse and builds trust in those data resources **[R7]**. This requires funds to sustain the expert interpretation and classification of vast amounts of information (Bourne *et al*, 2015; Karp, 2016; Chen *et al*, 2020). Expert curation is essential for the provision and dissemination of high‐quality, accurate data and the associated metadata, which can be productively used for specific research applications. **Delivering early and efficient curation** processes that reduce human effort through automation is a challenge for sustainability; rewards can be efficient incentives **[R10]**. Large‐scale data curation can be supported by specific biomathematical models used to process and integrate big datasets programmatically, e.g. JWS Online (https://jjj.biochem.sun.ac.za/) and EBI BioModels (https://www.ebi.ac.uk/biomodels/). These approaches often help the discovery of inconsistencies among the datasets, in terms of biological, physical, chemical or even mathematical incongruities. The registration, dissemination and application of reference collections of curated **[R7]**, FAIR data repositories **[R5]** are primary goals of EOSC‐Life (Perseil *et al*, 2020 in Appendix 1; Box 3).

Box 3 **Collection and resource management.**

Some of the collections described by open standards are provided in support of EOSC‐Life Demonstrators and Open Calls projects (Parkinson *et al*, 2021, 2022 in Appendix 1). For instance, the PDB‐REDO resource (EOSC‐Life Open Call project) exploits collections of curated crystallographic datasets to refine, rebuild and validate structural models of biomolecules automatically (Joosten *et al*, 2014). An extensive collection of open standards and data portals is now available to LS communities. To facilitate discovery and use, these resources are registered in FAIRsharing (https://fairsharing.org/).

**FAIRsharing collection and EOSC**

Embedded in EOSC, and recommended by funders and publishers, FAIRsharing is a curated, informative and educational resource for data and metadata standards that is interrelated to databases and data policies and extends across all disciplines (Sansone *et al*, 2019). FAIRsharing encourages users to discover, select and exploit these resources with confidence. It also encourages producers to make their resources more sustainable and discoverable, so that they will be more widely adopted and cited. In the context of the sustainable findability of EOSC‐Life aligned outputs, a dedicated collection of its 133 data resources are richly described. The descriptors used are also served by FAIRsharing in a machine‐readable form to feed the information into EOSC portals and other tools, such as the OpenAIRE Graph (Appendix Supplementary Information S9). Many of the descriptors themselves (e.g. life cycle status, links to sustainability documentation and relationships to the organisations who fund and maintain them) are also key to helping both EOSC and the wider research community assess the sustainability of the resources curated within FAIRsharing.

#### Provenance for sustainable use

Provenance provides the history of an object. Thus, it can be used to assess an object's quality, reliability, usefulness and trustworthiness, which are all important for its re‐use (Moreau & Missier, 2013). Providing the provenance itself has to be technically and organisationally sustainable (Box 4).

Box 4 **Key aspects for ensuring sustainable provenance (Traceability of Data).**

Provenance information is always provided via metadata (**who, what, where, when and how**) and often consists of heterogeneous data, such as software logs, workflow input files and Standard Operating Procedures. As such, the steps that need to be taken to ensure the sustainability of the provision and the existence of the (meta)data apply equally to the provenance (meta)data. To achieve this sustainability, the following are needed and, in turn, should themselves be sustainable resources:

- **Standards**, e.g. for metadata and data formats, vocabularies, provenance and workflow management and security.
- **Tools for accessing provenance information**, such as the LS AAI, OLS and FAIRsharing.
- Suitable and continually **relevant access technologies and methodologies** for human and machine consumers.
- **Accessibility via online archives/portals**; the adoption of policies concerning how long provenance (meta)data are stored and shared and how long the necessary AAI (especially for sensitive information) is provided, updated and versioned.
- Appropriate **cryptographic techniques**, e.g. hashes and digital signatures, used in combination with security policies.

EOSC‐Life has facilitated the development of the Common Provenance Model (Wittner *et al*, 2022). It introduces a framework to handle distributed provenance information **[R5]**. Such a framework has already been applied in the BY‐COVID (https://by‐covid.org/) project, and its use in other projects, including in BIOINDUSTRY 4.0 (https://cordis.europa.eu/project/id/101094287), is planned. The model also serves as a conceptual foundation beyond open science communities as it is being used for the proprietary ISO 23494 (https://www.iso.org/standard/80715.html) provenance standard series, currently being developed to provide a standard **sustainability framework** for the biotechnology industry.

The same principles apply to computational work, where the use of modern workflow management systems provides a “retrospective provenance”: the detailed record of the implementation of a computational workflow with information related to every executed process and the execution environment used (Khan *et al*, 2019). In this context, the FAIRification of software, which is often initially conceived as a stand‐alone tool, would benefit from the software also being designed for inclusion in workflows (Brack *et al*, 2022). The goal is to develop computational workflows that can be easily used with modern workflow management systems, as well as be retrieved and deployed seamlessly from registries such as the WorkflowHub (Goble *et al*, 2021, 2022 in Appendix 1), while remaining, evolving and being curated in their home repositories **[R7]**. Workflows become more sustainable thanks to their portability across hosts and the reduced deployment overhead for new users that such workflow management systems provide. Two main EOSC‐Life contributions to the management of provenance are the systematic use of RO‐Crate (Soiland‐Reyes *et al*, 2022) and LifeMonitor **[R9]**. The latter service complements the WorkflowHub by ensuring the correct operation of the workflow over time, through monitoring and then triggering automated workflow tests.

#### Traceable legal requirements from the data

Data that were collected from physical sources, e.g. human subjects, lab animals or field samples, for which legal and policy steps of any type were required, are not by definition usable, unless these steps are documented. In different cases, sustainable access to the original or derived data must be ensured. Proof of compliance with legal requirements is part of the provenance of data **[R5]**. Clear governance approaches for data use are needed to ensure that the investment in data generation is matched by sustained usage and to ensure that relevant laws are adhered to **[R8]**. For controlled access data, such as human genetic data, GA4GH standards can be referenced to annotate each data use case and provide a machine‐readable terminology (Lawson *et al*, 2021) that can be implemented by multiple resources. EOSC‐Life has contributed to the development of these standards, as well as operationalisation of the standards, e.g. through development of the LS AAI system to support machine readable access protocols (“GA4GH passport and visas”, Cabili *et al*, 2021).

Sustained usage and FAIR implementation require machine‐readable (meta)data and software licences. We have found that the more open the licence, the more likely that the data or software will be reused. EOSC‐Life has promoted technical sustainability by using permissive licences (https://fossa.com/blog/all‐about‐permissive‐licenses/) and open‐source codebases, (https://pncnmnp.github.io/blogs/oss‐guide.html) which can be reused and receive contributions from a wide community.

#### Role of cloud providers

In the past, widely accessible services were designed and deployed from a central location. As cloud deployment costs have decreased, and because some datasets/data resources are deployed in industrial and secure settings, the interoperability services must also be portable. This offers software scientists considerable benefits, as portable resources are more agile to be developed with multi‐site teams **[R6]**, and their deployment is often simpler, resulting in a reduction in the overall effort to sustain a service.

To enable the deployment of LS workflows in the EOSC, EOSC‐Life relies on a technology stack that standardises the software installation process to ensure the reproducibility of computational workflows and automates software deployment in cloud environments. The key to community adoption has been the availability of free and publicly accessible services and technologies **[R11]**. Integrating such free‐to‐use services and technologies has made the research process more efficient, reproducible and collaborative, but has also resulted in some sustainability and reproducibility risks. Currently, components of the computational ecosystem rely on services from commercial entities. For example, we estimate that GitHub (https://github.com/) provided services to the bioinformatics community in 2021 with a value of over $1 million. Similarly, quay.io (https://quay.io/) provides services with an estimated $500,000/year in value for BioContainers (https://biocontainers.pro/). Communities work on the assumption that the conditions under which these technologies and services are provided will remain compatible with the research requirements and the companies' abilities, e.g. that they will remain free of charge and freely accessible, and have adequate performance characteristics. This may not always be true (Box 5).

Box 5 **Code and software management.**

Already, over the project's lifespan, we have seen changes in and restrictions to some widely used services. To highlight a few:

- **TravisCI** (https://www.travis-ci.com/): a popular continuous integration system, initially used by the LifeMonitor, switching to a paid model.
- **DockerHub** (https://hub.docker.com/): a well‐established repository of container images, which introduced limits to the number of containers that can be pulled without subscription.
- **Conda** (https://docs.conda.io/en/latest/): a popular open source package management and environment management system which changed the licence terms of its default channel usage.

Code repositories are particularly at risk, as it has been demonstrated in the past by the disappearance of Google code (2006–2016) and the bundling of junkware/malware by SourceForge (https://sourceforge.net/) with each download, in an attempt to become profitable. Larger institutions already run their own code‐hosting platforms, but most RIs do not have sufficient IT infrastructure or human resources in place to do so.

#### Sensitive data challenges

**Figure 3:**
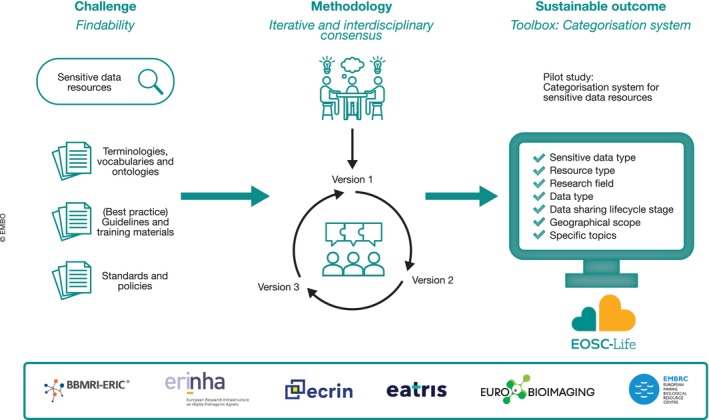
EOSC‐Life Sensitive Data toolbox development scheme One main challenge regarding sensitive data concerns the findability of resources and terminologies used to classify data. An iterative method, involving the subset of the participating LS RIs (BBMRI (https://www.bbmri-eric.eu), ERINHA (www.erinha.eu), ECRIN (https://ecrin.org/), EATRIS (https://eatris.eu/), EUROBIOIMAGING (https://www.eurobioimaging.eu), EMBRC (https://www.embrc.eu/)), with several versions, was used to reach an interdisciplinary consensus on a sustainable categorisation system, and the classification of toolbox resources by sensitive data type, resource type, research field, data type, stage in life cycle, geographical scope and specific topics related to sensitiveness.

Across research domains, sensitive data present challenges related to sustainability, cross‐domain categorisation and discovery. Sharing sensitive data within EOSC‐Life is particularly challenging when they need to be made available to third parties not contractually bound to the original data controller. The sensitivity of the data may arise not only from their personal nature but could also originate from intellectual property considerations, biohazard concerns or compliance with the Nagoya Protocol (https://www.cbd.int/abs/). With regard to Access and Benefit‐sharing (Appendix Supplementary Information S7), and as sharing may be perceived as a risk, EOSC‐Life has developed a prototype toolbox (Ohmann *et al*, 2022) allowing researchers to find existing, reliable resources relevant for sharing sensitive data across all participating RIs (Appendix Supplementary Information S8), supported by an interdisciplinary categorisation system **[R9]** (David *et al*, 2022) for which sustainability is increased by iterative building, validated by iterative consensus (Fig 3). Another example, the Open‐Source Secure Data Infrastructure and Processes Platform (https://www.ifs.tuwien.ac.at/infrastructures/ossdip/; OSSDIP – Weise *et al*, 2021) was developed as a blueprint that can be deployed, e.g. for educational purposes **[R11]**. OSSDIP clarifies the rather complex underlying concepts and reduces the initial burden of deploying such an infrastructure, giving researchers access to sensitive data in return.

#### Skills and capabilities to design, setup and implement

Systematic assessments have shown that both individual projects and RIs display a broad range of maturity levels, in terms of their cloud implementation status, and the technical skills of team members involved in the deployment and management of deployed resources. To address existing gaps, and support the development of organisational expertise **[R1]** to sustain long‐term operation, as described below (Part 3 and **[R4]**), training processes and materials were established in EOSC‐Life. Having appropriately trained and rewarded personnel not only benefits the RI but also provides individuals with career advancement opportunities **[R10]** and transferable skills which, in turn, support innovation **[R12]** and economic development.

### PART‐3: driving and sustaining the EOSC‐Life community and expertise through training and skill development

Training initiatives were designed to promote community development and cross‐RI collaboration, as well as to help LS RIs meet the technical challenges of sustainable FAIR implementation (David *et al*, 2020) with respect to both the data and training materials themselves (https://www.eosc‐life.eu/services/training/). These initiatives aligned with our aim to promote a cultural change, encouraging users to adopt Open‐Science practices. **Training is a community driver**, and not only provides technical expertise enabling researchers to improve the handling of their data (e.g. data management, analysis, annotation) in a sustainable manner but also reinforces the expertise. Supported by a dedicated training team in EOSC‐Life, collaborative training activities were delivered by multiple consortium partners, and RI experts, permitting cross‐domain, cross‐RI training programmes for diverse user needs **[R4]**.

Domain‐specific training activities were also promoted and supported through the “Training Open Calls” (Box 6).

Box 6 **BioImage data analysis training supported by EOSC‐Life open calls.**

The NEUBIAS training school (May 2023; https://www.eosc-life.eu/news/neubias‐defragmentation‐training‐school/) provides one example of RIs benefiting from the EOSC‐Life Training Open Call. This course was the continuation of an initiative that started in 2017, aiming to bring BioImage Data Analysts closer to recent solutions for computing and workflow‐based image analysis in the cloud. For the NEUBIAS (https://eubias.org/NEUBIAS/venue/) project, EOSC‐Life support has been essential for sustaining, further developing and running this valuable training initiative.

Image‐based data are ubiquitous across multiple LS‐RI domains. Because of the multimodality and the large volumes of data, linkage of image data to publication analysis workflows and the sustainability of access and hosting are key ongoing challenges. Training in this field has been provided regularly through EOSC‐Life partners and project leads, including FAIR training from European Molecular Biology Laboratory (EMBL)‐EBI (Image Analysis and Machine Learning; https://www.ebi.ac.uk/training/events/microscopy-data-analysis-0/) or FAIR Data training run by national and international consortia, e.g. Euro‐BioImaging (https://www.eurobioimaging.eu/news/euro-bioimagings-guide-to-fair-bioimage-data/; Kemmer *et al*, 2023).

Overall, 15 training activities (https://www.eosc‐life.eu/services/open‐call‐training/) were established, which benefited 13 LS RIs and 27 user projects. The training calls contributed to the strategic effort to link the work of EOSC‐Life to each RI's development plans, improve the visibility of ongoing work on RI‐leaded resources and address gaps in expertise by carrying out specific activities and capacity building (Box 7) **[R1]**.

Box 7 **Sustainability of knowledge through EOSC‐Life training assets.**

By supporting cross‐disciplinary sharing of best practices and methodologies, the consortium contributed to sustainability of knowledge across LS communities through:

- Training on EOSC‐Life and RI **cloud‐based open access** solutions and resources, services and expertise in the dissemination, practical uptake and targeted adoption of tools.
- Training related to **Open Science** practices and **FAIR guidelines**, harmonising the requirements across a spectrum of LS disciplines whilst addressing needs in individual branches.
- Establishment of **expert groups** to provide guidance and consultation to broader communities within the framework of Open Science and FAIR Science, resulting in a network that can continue operating after the project.
- Development of collaborative **cross‐disciplinary training approaches**, modules and materials, as well as formats for effective training seminars and workshops, including the joint creation of best practice solutions for efficient remote training.
- **Integration of 27 Demonstrators**, namely Open and Internal Calls user projects from different disciplines, into EOSC‐Life and across the RI landscape by providing consultation and guidance for the teams in the form of advice from EOSC‐Life experts and consortium partners.
- Designation and training of the so‐called EOSC‐Life **consortium translators**, to create a cohort of professionals that understand the jargon, needs, work culture and drivers of professionals working in other areas of expertise or RIs.
- Creation of a **training community** with a practical understanding of EOSC but also with conceptual and technical knowledge in different LS domains.

To speed up the evolution of the RIs, and the integration of user projects, we employed consultative orientation meetings and hackathons, including the cross‐RI FAIR Hackathon for Training on Demonstrators, and Open and Internal Calls project teams. This consortium‐wide training was provided to **assess and improve the FAIRness** of the projects, orient the teams based on available examples, share knowledge on solutions and resources provided by EOSC‐Life experts **[R1]** and RIs communities. Overall, the initiatives provided fruitful opportunities and platforms for goal‐oriented, hands‐on teaching, introducing EOSC‐Life topics to consortium teams. Such interactive platforms for consortium‐wide exchange facilitated further networking and collaboration among experts from the EOSC‐Life community and strengthened capability building in new projects (Example in Appendix Supplementary Information S10).

This consortium effort in building up knowledge, skills and trust, made possible rapid responses to large‐scale scientific, societal, environmental or other challenges of concern, e.g. pandemics similar to the COVID‐19 pandemic **[R6]**. A compelling EOSC‐Life example for implementation of interdisciplinary training, to address challenges that emerged during the coronavirus pandemic, was the creation of an epidemiology mathematical modelling training course (https://www.eosc‐life.eu/news/training‐modelling‐covid‐19‐epidemics/), containing content relevant to future pandemic situations.

EOSC‐Life's investment in **building a community of experts**, and in providing training to these experts, has already increased organisational and technical sustainability. It is hoped that experts trained in the EOSC‐Life consortium will continue working in RIs, taking on the role of master trainers, disseminating their expertise to others. To promote and strengthen the evolution of the cross‐RI expert network and support cross‐community activities, EOSC‐Life also organised the “EOSC‐Life translator training series” **[R9]**, which enables the experts to understand the drivers and challenges in the different RIs and data professions and break down silos **[R1]**.

## Discussion

### Technical and operational sustainability

The long‐term preservation of data and tools for re‐use currently relies on multiple repositories. In such a fragmented landscape, sustainability depends on broadly distributed funding, but also on adequate linkage and/or cross‐referencing between repositories. This is becoming a pressing issue for multi‐modal datasets, where individual components are split into data‐type specific repositories. Examples of this are single‐cell omics datasets combining imaging and sequencing, where the sequencing data are submitted to sequence archives, and images are submitted to imaging archives, without necessarily any formal linkage. The wider adoption and implementation of linked data principles (https://www.w3.org/DesignIssues/LinkedData; Bizer *et al*, 2009) could mitigate this. Fragmentation of a project's data also prevents users from accessing them as a whole and fully understanding them. One way forward could be to build dedicated web applications and API access allowing the visualisation and browsing of project‐specific data on top of distributed data archives. While some research groups already implement this in their projects, such web applications have tended to have a limited lifetime.

The use of cloud resources in EOSC‐Life has been limited to well‐established academic clouds mainly due to associated costs. A sustainability threat due to the use of a commercial cloud arises from, on the one hand, the expertise gap created by outsourcing and, on the other hand, a lack of cost control due to vendor lock‐in, which is seen as incompatible with the time limited, fixed budget of most research grants. In the case of sensitive data, the complex legal framework and country derogations means that most institutions are risk averse and researchers refrain from using cloud environments that are not directly under their control.

**Validating the adaptability of sustainability components** developed in EOSC‐Life for use in other projects is key to ensuring the project's long‐term impact. The recent EC's INFRASERV (https://rea.ec.europa.eu/funding‐and‐grants/horizon‐europe‐research‐infrastructures/research‐infrastructure‐services‐support‐health‐research‐accelerate‐green‐and‐digital‐transformation_en) programmes represent both a significant challenge to models of sustainability for FAIR LS resources and an opportunity to create more impactful cross‐RI initiatives. By focusing on ISIDORe, (https://isidore‐project.eu/) which is centred on pandemic preparedness (Richard *et al*, 2022; David *et al*, 2023b), to provide an INFRASERV perspective on the topics raised in this paper, we showed the critical importance of improving FAIRness Literacy, setting up incentives, training researchers and providing FAIR expert support for sustainable data management **[R1]**, **[R4]**, **[R5]**, **[R10]** (Appendix Supplementary Information S11).

### Financial sustainability

Open‐Science allows collaborations to take place without the red tape of negotiating access, intellectual property transfer and detailed assignment of ownership. Thus, Open‐Science practices are not only effective in bringing people together around common solutions but are also unparallelled tools for long‐term sustainability in a complex landscape of national and international funding sources **[R11]**.

**Figure 4:**
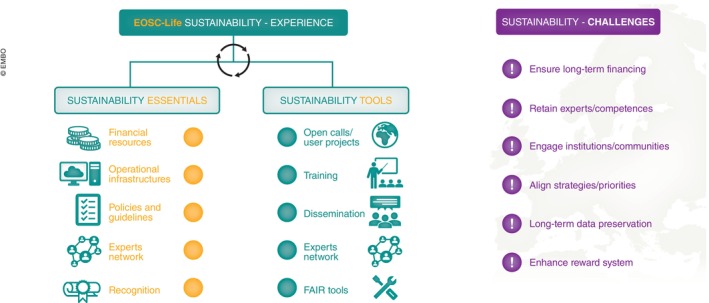
Key component driving, facilitating and challenging the sustainability process—lessons from the EOSC‐Life project **Sustainability essentials** are prerequisites including availability of financial resources, competent experts, technical infrastructures, aligned policies and recognition. These components allow a set of **sustainability tools** operating towards sustainability to be defined: e.g. via integration of user projects (open calls); community training; dissemination addressing specific needs; creating and expanding the experts networks. The main linked **sustainability challenges** are securing long‐term financing to retain expertise and maintain solutions; engaging institutions and communities while harmonising alignment in common strategies and priorities; and enhancing a reward system that supports re‐use of available resources.

The network of experts across the RIs and LS domains established as a result of EOSC‐Life provides a solid foundation for ensuring the sustainability of knowledge, the reusability of EOSC‐Life tools and resources and the creation and promotion of EOSC services, increasing adaptability for emerging user needs. These experts, e.g. data stewards who have domain‐specific skills, and a firm understanding of Open‐Science Cloud solutions, are key to technical and operational sustainability within a FAIR and Open‐Science framework (Fig 4). Setting aside the question of the acute lack of qualified personnel, the activities and disponibility of these professionals, with their unique skill sets, can only be sustained by ensuring adequate financial resources. This would allow stable employment and keep cloud infrastructure solutions operational.

**EOSC‐Life** does, however, face a general sustainability challenge in that it relies almost completely on EC project funding. It is thus dependent on the **integration of its outputs into new EC proposals** and initiatives. These are constrained by their own limited lifespans and the need to focus on constant innovation and new developments, rather than on the operation of existing assets. This in turn can have a negative impact on research, as it can be easier to obtain funding to reinvent services and resources, rather than to support the long‐term operation of existing valuable resources, and their experts (EOSC TF FinSus, 2022).

While, in the case of EOSC‐Life, some resources have already been taken up by other projects, for many of its resources, there is still no immediate follow‐up funding available. This means the project partners need to find alternative financial means to sustain the resources, as well as the respective expertise. In essence, the EOSC‐LIFE project partners have two **options**:

- A partner organisation **takes it on as a core resource** or
- the resource **becomes the responsibility of a community**, i.e. the means to operate, maintain and develop the resource is given through future grants and/or “in kind” contributions from intrinsically motivated research parties/individuals (in an equivalent model to various open‐source communities).

It is important to note that, in either model, full responsibility not only includes the continued technical and operational support for the resource but also the curation of the resource, which ensures that the content is correct and up to date.

There is a risk, however, that if no project partner takes on the responsibility, and no immediate effort is made by the community, awareness of an existing tool can be lost. This leads to resource‐intensive reinvention of something similar; it may take months, sometimes years before a tool is taken up again. For instance, the project Biotracks (https://github.com/CellMigStandOrg/biotracks) provides a standard format for cell migration files and a series of converters that can be used to convert popular tracking software packages to the Biotracks formats. The project was supported by CORBEL (https://www.corbel‐project.eu; 2016–2020). Due to the lack of funding, the project was put on hold. It is only now that the project has been revived as the need for re‐use has emerged in the context of Open Microscopy Environment Next‐Generation File Formats (https://ngff.openmicroscopy.org; Moore *et al*, 2021).

To address this issue before it became urgent, the EOSC‐Life open calls included a consultation process to advise the many small teams (typically two to five members per participating institution) who were applying. As part of the project submission process, experts from EOSC‐Life **analysed the needs of each team and provided a roadmap** with recommendations to ensure successful project implementation, even if the funding application was unsuccessful. The applicants acknowledged the utility of the consultation process, and it helped several teams improve FAIR data management strategies that could be adopted even in absence of funding (Rybina *et al*, 2023 in Appendix 1). This process is, however, resource intensive and relies on the availability of experts to provide consultations and the relevant training materials **[R1]**, **[R4]**.

Clearly, reinventing the wheel or developing new solutions inferior to existing ones may not be the best use of funding resources. There are **four critical phases** of resource development requiring specific financial support: **conception, proof of concept development, stabilisation and community adoption** with long‐term sustainability, dissemination and maintenance **[R6]**. It is challenging to explain to funders and policy makers that the last two are crucially important **[R12]**. But until there is recognition that maintaining existing resources is critical and should be a priority for investments in RIs, maintenance will continue to rely on volunteer efforts and in‐kind contributions, solutions that are clearly not sustainable.

Therefore, to guarantee sustainability for successful project outcomes, individual RIs need to engage early on in any project with national and European funders, as well as through existing stakeholder networks such as the LS RI Strategy Board and ERIC Forum **[R11]**, to present clearly and repeatedly the outcomes and messages outlined in this paper **[R2]**.

## Recommendations

**Figure 5:**
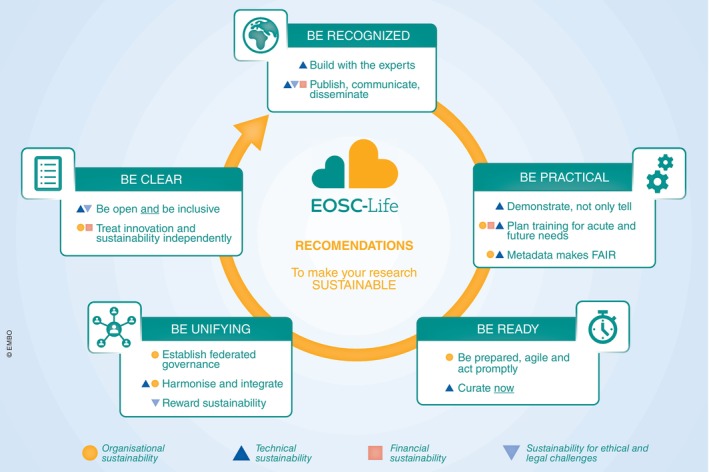
EOSC‐Life Recommendations for ensuring and facilitating sustainability Recommendations for ensuring and facilitating sustainability are based on 5 pillars shown here, clockwise from the top: to base the network on experts, and disseminate on a broad‐scale; and to demonstrate, to act and plan training and improve metadata; to be prepared to act now, but remain adaptable, and curate data as soon as possible; to strengthen the community with federated governance, harmonised and integrated RIs, and sustainability actions rewarded; to be inclusive and open‐minded, and to treat innovation and sustainability independently.

Not all data infrastructures can or should be supported indefinitely. Financial sustainability is achieved when funders (public or private) are willing to **prioritise necessary long‐term investments**, based on the expectation that **impact**, in terms of **science** and innovation, **can be achieved**, but also that **societal threats** can be tackled, such as pandemics and other disasters. While the COVID‐19 pandemic has proven the value of LS data and models for practical assessments and policy decisions, their wider potential still remains largely under‐exploited. More efforts are needed to broadcast the many ways in which LS data can be used and to help government representatives, journalists and members of the general public understand LS data. Therefore, new activities may be necessary that not only ensure that these data are sustainable but also that they are impactful and considered as indispensable. All the work and experiences collected during EOSC‐Life allow us to propose the following list of recommendations (Fig 5).

**BE RECOGNISED**: Focus on **strong credibility and recognition** for the research done:

- **[R1] Build with the experts**: The EOSC‐Life open calls demonstrated that a consultative process, providing expert advice to teams before they submitted an application, resulted in improved adoption of FAIR data management strategies, irrespective of whether the application was funded or not. Keeping in mind the limited number of available experts and training materials, this approach could help future projects with limited funding options find ways to adjust their resources accordingly.
- **[R2] Publish, communicate, disseminate**: Effective communication through dissemination to funders that relays the impact of previous investments and the benefits of continued support is crucial for achieving long‐term alignment between funding decision‐making and the development plans for RI data/software/workflow resources. In addition, raising awareness for available and accessible tools and resources, i.e. disseminating and sharing FAIR solutions with a broader audience, and increasing their reusability, impact and overall sustainability, are key. Furthermore, peer‐reviewed publications, preferably in Open Access journals, remain the main way to sustainably disseminate recognisable and findable statements and research. New publication types, efficient for dissemination and reusability, are emerging for all Digital Objects sustainability, as they are for data, workflows and softwares.

**BE PRACTICAL: Demonstrating, practising and reproducing** permit better and **sustainable adoption** of research outputs:

- **[R3] Demonstrate, not only tell**: EOSC‐Life has shown that establishing FAIR resources that meet the needs of parallel LS communities creates the necessary scale to drive financial and operational efficiency and reduce fragmentation. The EOSC‐Life strategy of learning by doing, based upon addressing the concrete needs of scientific demonstrators, helps funding organisations better understand the inherent scalability and translatability potential of our approach.
- **[R4] Plan training for acute and future needs**: Developing competent, effective and sustainable training methodologies and formats within the scope of an inclusive cross‐disciplinary Open‐Science framework is a stepwise, evolutionary process. It requires (i) connecting experts, trainers and users in a goal‐oriented manner, such as through open calls, (ii) creating adaptive platforms with resources that promote targeted dissemination and the exchange of knowledge, enabling a rapid response to the needs of different communities and (iii) providing sufficient sustainable funding schemes to keep this network of experts active and functional beyond time‐limited tasks. Making investments in sustaining competent human resources and training is essential to ensure the uptake of tools, services and solutions and to integrate other expertise and diverse communities.
- **[R5] Metadata makes FAIR**: Repositories or portals hosting scientific digital objects in a semantically interoperable manner or collections of physical material (biobanks, culture collections) that provide sufficient provenance and other FAIR (meta)data with the objects they share are becoming more trusted sources of scientific resources, promoting their use in the community. For this reason, guaranteeing provenance is recommended to ensure the practical sustainability of scientific objects and the services that provide them, i.e. allowing sustainability through re‐use.

**BE READY**: Sustainability requires **agility and readiness to catch opportunities**:

- **[R6] Be prepared, agile and act timely**: Agility is often forgotten but is required to address emerging challenges, and this is particularly true in the LS. EOSC‐Life has applied its action plan, which has a high level of adaptability, in order to be able to anticipate and provide non‐centralised services and expert support during the COVID‐19 crisis. To ensure sustainability, we must proactively take advantage of all relevant opportunities. One key lesson learned through the EOSC‐Life Open Calls was the high importance and value of facilitating interactions and regular exchange between domain‐specific experts and project teams. This approach triggered an early uptake of available tools and knowledge and also activated effective, collaborative and timely co‐development of targeted solutions and guidelines, spreading across disciplines.
- **[R7] Curate NOW**: EOSC‐Life has invested in projects which prioritise, leverage and extend expert curation processes. These projects build capacity, adding value to existing data and delivering processes which can be re‐used and combined with automated techniques for data annotation. The provision of high‐quality, curated datasets is also essential for the development of new AI and data‐driven initiatives and projects in LS research. Assessing the efficiency of quality processes could help to improve research outputs.

**BE UNIFYING**: Sustainability of products is **based on clearly identified, recognised and driven communities**:

- **[R8] Establish federated governance**: The long‐term responsibility for the sustainability‐related activities must be clear from the outset. Responsibilities and roles that ensure sovereignty and access to sustainable services and tools must be organised during the implementation of research activities. Therefore, the provision of adequate funding and planning is vital, particularly concerning semantic interoperability and data quality.
- **[R9] Harmonise and integrate**: It is easier to encourage the long‐term adoption of new operating systems by potential users when these can be connected with previously issued products, and if these are backwards compatible with previously issued tools, scripts and software. Harmonisation, reached by iterative consensus, facilitates and guarantees the interoperability of tools, data and solutions and improves the understanding of concepts, functionalities and semantics shared across communities and disciplines. It is also easier to maintain services if they are fully integrated into the relevant communities' ecosystems.
- **[R10] Reward sustainability**: Further efforts and investments of resources devoted to increasing sustainability are often considered as unprofitable in the short term. They should, however, be promoted and rewarded as a means of increasing the quality of products and when they are recognised by users as being essential. Rewarding contributors for their extra efforts and making them visible in the community contributes to success and cohesion.

**BE CLEAR**: Inclusiveness and openness, as well as innovation, sustain communities:

- **[R11] Be open and be inclusive**: Open specifications of data formats and data exchange protocols promote trust and inclusiveness and safeguard against obsolescence. Inclusiveness is critical to maintaining interest, increasing adoption and integrating newcomers who can sustain and reuse solutions and tools. Community building is a key component for long‐term training, good data‐sharing literacy and efficient project output dissemination and reuse.
- **[R12] Treat innovation and sustainability independently**: Our recommendation for funders is that they recognise that sustaining the operation of a resource is an activity in its own right. For this reason, they should encourage the reuse and expansion of existing resources and facilitate the development of innovative tools and services. Consultation among stakeholders when developing funding calls can ensure alignment between the funders' aims and the community needs.

**Figure 6:**
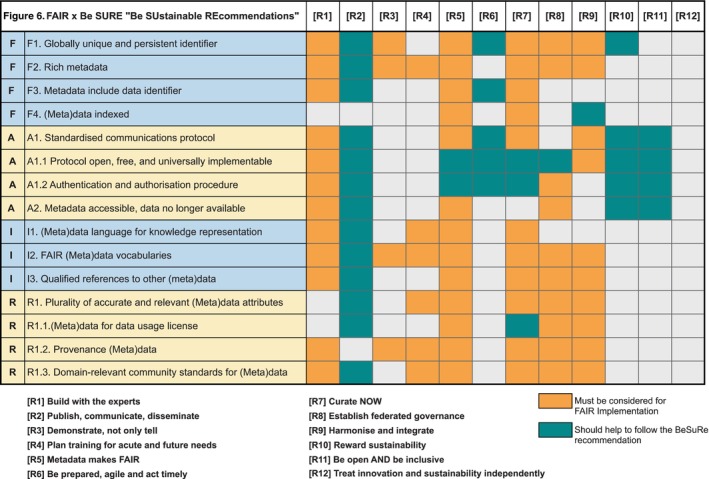
Cross‐Be SUstainable REcommendations and FAIR principles implementation Classification of EOSC‐Life “Be SUstainable REcommendations” in essential recommendations (orange) and recommendations which can be of help (green) for concrete FAIR principles implementations.

Even if many of these sustainability recommendations seem to be obvious, the practical understanding and actual implementation may be challenging especially when its importance and overall impact on short and long‐term perspectives are poorly understood from the start of a collective community work. The recommendations provided can, however, be explicitly and bidirectionally linked to the FAIR principles and concrete processes related to data and tools handling, sharing and reproducibility. This approach was also taken by the IMI FAIRplus project (https://fairplus‐project.eu/; Rocca‐Serra *et al*, 2023; Welter *et al*, 2023), which demonstrated the value of FAIR principles implementation by engaging with and FAIRifying data resources from 20 research consortia. By adopting such FAIRification actions, we are able to classify the key objectives **which should help** in FAIR implementation (that reciprocally help the sustainability research outputs), **or are mandatory to permit the quality, the efficiency and the sustainability** of FAIR compliance (Fig 6 and Box 8):

Box 8 **How our recommendations support (and are supported by) sustainable FAIR principles compliance.**

Most of the FAIR principles (F1, F2, A2, I1, I2, R1.1, R1.2, R1.3) strongly depend on an initial “agreement”, at a minimum at the domain level (Jacobsen *et al*, 2020). **Expertise support** [R1] is a pillar for improving the implementation of each principle and for the planning, support and design of training as well as for FAIRness assessment. Additional pillars important for recommendations in the sustainable implementation of the FAIR principles are: the organisation of **sustainable community building** [R9, R11] and the **governance and acknowledgement of community agreement processes** [R8, R10].

**“Metadata makes FAIR”** [R5] is the key component for the application of FAIR principles. Sustainable data sharing and reuse supported by appropriate and sufficient metadata must be *on the one hand* explained and driven by community experts and data stewards [R1], and, *on the other hand*, illustrated through **real case studies** [R3], **implemented iteratively through hands‐on training** [R4] and adapted taking into account **specific and arising needs of communities** [R9]. (Meta)data **annotation and curation** [R7] are also essential to ensure (i) informative and rich metadata (F2, R1), especially when specific to a particular research area (R1.3), (ii) relevant, interoperable and updated vocabularies (I1, I2) and (iii) cross‐domain qualified references to other (meta)data (through interoperable metadata; F3, I3) enhancing semantic harmonisation and convergence and helping build the *Web of Linked Data*.

**Communication through publications** [R2] helps promote, multiply ways, diversity and opportunities of linking, reuse, and naturally accelerates the understanding of (meta)data. The publication of several types of metadata should be prioritised to allow effective and fruitful dissemination (re: [R2]). This is a central step in the implementation of the FAIR principles (F2, F3, and finally F4). The “metadata indexation” principle (F4) is, in our experience, often omitted or its importance misunderstood.

Our recommendations also promote the improvement of the (current) practices around reuse of data and tools: the **Openness recommendation** [R11] advocates the usage of open licences, preferably CC‐0 or CC‐BY ones, for ease of reuse (R1.1). Providing provenance metadata and provenance tracking methodologies (R1.2), especially in complex information systems, requires **early preparation and curated recording** [R7], adequate metadata (F2, R1) and pre‐validated (FAIR) interoperable, unambiguous vocabularies (I2). Our recommendation [R6] **Be prepared, agile and act timely**, also highlights and underlines the importance of applying FAIR principles as early as possible.

Even if some of the recommendations may not be directly linked to the FAIR principles (e.g. A1—A1.1, A1.2: *open free access protocol*, or A2: *metadata are accessible even if data are no longer available*), systematic FAIR implementation will be encouraged by **dissemination of efficient tools and good practices** [R2], and **well‐governed reward mechanisms** [R8, R10].

The recommendation [R12] could be interpreted as “**Treat innovation *and FAIR principles* independently**” and to consider the FAIR implementation as an evident part of sustainability. The FAIRification process, even more through “data sharing” will most likely create new collaborations, especially between cross‐disciplines, therefore facilitating and enhancing innovation. Finally, we would like to stress that the implementation of all the **FAIR principles are necessary for and will catalyse action on**, the 12 *Be SUstainable REcommendations “Be SURE”*.

## Conclusion

The EOSC has formed “a federated and open multi‐disciplinary environment where users can publish, find and re‐use data, tools and services for research innovation and educational purposes” (https://www.eosc.eu/sria‐mar). Through the EOSC‐Life project, we set out to create an “EOSC for the Life Sciences”, connecting, and where necessary, further developing data resources, analysis tools and services that allow research communities to collaborate across national and thematic borders. A core part of the project and its sustainability strategy was to work in close partnership with the broader life‐science community, via open calls for partnerships. This strategy has been successful. As EOSC‐Life comes to its end, the services that emerged are being carried forward by a range of applied projects: BY‐COVID is consolidating an Open‐Science platform for pandemic preparedness; EOSC4Cancer (https://eosc4cancer.eu/) will adapt several EOSC‐Life solutions for use by the Cancer Mission; and EuroScienceGateway (https://galaxyproject.org/projects/esg/) will continue to develop the tools and software ecosystem initially developed in EOSC‐Life together with experts from the earth, environmental and physical sciences.

EOSC‐Life has thus helped the life sciences to take significant steps towards turning the EOSC vision into a reality, but much work remains to be done. How can our experiences shape the future development of EOSC and the data environment for Europe's LS RIs? Even if EOSC‐Life underlines the importance of close partnerships, and open calls are becoming even more challenging to implement than pre‐defined use cases in our experience, these calls are critical for developing and advancing practical applications driven by user needs and for promoting scientific discoveries. Future EOSC developments should build on the capacity of whole‐domain projects such as EOSC‐Life (and the four other European research clusters), which serve as a nucleus for managing data and connecting interdisciplinary communities. In addition to the INFRASERV projects mentioned above, canSERV (https://www.canserv.eu/) connects experimental facilities that support the analysis of biomedical data in cancer. Other projects are promoting the agroecological transition (AgroServ, https://emphasis.plant‐phenotyping.eu/european‐infrastructures/cluster‐projects/agroserv) and supporting AI‐powered image analysis methods (AI4Life, https://ai4life.eurobioimaging.eu). All of these projects build on the tools and experiences supported by EOSC‐Life, including RO‐Crate, ISA tools (https://isa‐tools.org/), FAIRsharing and the FAIR Cookbook. These will continue to populate EOSC with data, workflows and other tools and to sustainably connect different disciplines.

Above all, EOSC‐Life has given a legacy in the form of the professional, intra‐ and inter‐disciplinary development of competences. The project has helped establish new data management capabilities in several RIs, and by providing training and support helped build skills in user communities. To be successful, EOSC needs to further develop networks of data managers and skilled data analysts throughout the European research community: Open data and Open Science can only generate value when the people are able to make use of the available opportunities.

## Author contributions

**Romain David:** Conceptualization; data curation; supervision; validation; investigation; visualization; methodology; writing – original draft; writing – review and editing. **Arina Rybina:** Conceptualization; supervision; validation; investigation; visualization; writing – original draft; writing – review and editing. **Jean‐Marie Burel:** Conceptualization; supervision; validation; investigation; visualization; writing – original draft; writing – review and editing. **Jean‐Karim Heriche:** Conceptualization; supervision; validation; investigation; visualization; writing – original draft; writing – review and editing. **Pauline Audergon:** Investigation; visualization; writing – original draft; writing – review and editing. **Jan‐Willem Boiten:** Investigation; writing – original draft; writing – review and editing. **Frederik Coppens:** Investigation; writing – original draft; writing – review and editing. **Sara Crockett:** Validation; investigation; visualization; writing – original draft; writing – review and editing. **Katrina Exter:** Investigation; visualization; writing – original draft; writing – review and editing. **Sven Fahrner:** Investigation; writing – original draft; writing – review and editing. **Maddalena Fratelli:** Investigation; writing – original draft; writing – review and editing. **Carole Goble:** Investigation; writing – original draft; writing – review and editing. **Philipp Gormanns:** Investigation; writing – original draft; writing – review and editing. **Tobias Grantner:** Investigation; writing – original draft; writing – review and editing. **Björn Grüning:** Investigation; writing – original draft; writing – review and editing. **Kim Tamara Gurwitz:** Investigation; writing – original draft; writing – review and editing. **John M Hancock:** Investigation; writing – original draft; writing – review and editing. **Henriette Harmse:** Investigation; writing – original draft; writing – review and editing. **Petr Holub:** Investigation; writing – original draft; writing – review and editing. **Nick Juty:** Investigation; writing – original draft; writing – review and editing. **Geoffrey Karnbach:** Investigation; writing – original draft; writing – review and editing. **Emma Karoune:** Investigation; writing – original draft; writing – review and editing. **Antje Keppler:** Investigation; writing – original draft; writing – review and editing. **Jessica Klemeier:** Validation; investigation; visualization; writing – original draft; writing – review and editing. **Carla Lancelotti:** Investigation; writing – original draft; writing – review and editing. **Jean‐Luc Legras:** Writing – original draft; writing – review and editing. **Allyson L Lister:** Investigation; writing – original draft; writing – review and editing. **Dario Livio Longo:** Investigation; writing – original draft; writing – review and editing. **Rebecca Ludwig:** Investigation; writing – original draft; writing – review and editing. **Bénédicte Madon:** Investigation; writing – original draft; writing – review and editing. **Marzia Massimi:** Investigation; writing – original draft; writing – review and editing. **Vera Matser:** Investigation; writing – original draft; writing – review and editing. **Rafaele Matteoni:** Investigation; writing – original draft; writing – review and editing. **Michaela Th Mayrhofer:** Investigation; visualization; writing – original draft; writing – review and editing. **Christian Ohmann:** Investigation; writing – original draft; writing – review and editing. **Maria Panagiotopoulou:** Investigation; visualization; writing – original draft; writing – review and editing. **Helen Parkinson:** Conceptualization; investigation; writing – original draft; writing – review and editing. **Isabelle Perseil:** Investigation; writing – original draft; writing – review and editing. **Claudia Pfander:** Investigation; writing – original draft; writing – review and editing. **Roland Pieruschka:** Investigation; writing – original draft; writing – review and editing. **Michael Raess:** Investigation; writing – original draft; writing – review and editing. **Andreas Rauber:** Investigation; writing – original draft; writing – review and editing. **Audrey S Richard:** Investigation; writing – original draft; writing – review and editing. **Paolo Romano:** Investigation; writing – original draft; writing – review and editing. **Antonio Rosato:** Investigation; writing – original draft; writing – review and editing. **Alex Sánchez‐Pla:** Investigation; writing – original draft; writing – review and editing. **Susanna‐Assunta Sansone:** Investigation; writing – original draft; writing – review and editing. **Ugis Sarkans:** Investigation; visualization; writing – original draft; writing – review and editing. **Beatriz Serrano‐Solano:** Investigation; writing – original draft; writing – review and editing. **Jing Tang:** Investigation; writing – original draft; writing – review and editing. **Ziaurrehman Tanoli:** Investigation; writing – original draft; writing – review and editing. **Jonathan Tedds:** Investigation; writing – original draft; writing – review and editing. **Harald Wagener:** Investigation; writing – original draft; writing – review and editing. **Martin Weise:** Investigation; writing – original draft; writing – review and editing. **Hans V Westerhoff:** Investigation; writing – original draft; writing – review and editing. **Rudolf Wittner:** Investigation; writing – original draft; writing – review and editing. **Jonathan Ewbank:** Validation; investigation; writing – original draft; writing – review and editing. **Niklas Blomberg:** Conceptualization; supervision; funding acquisition; validation; investigation; writing – original draft; project administration; writing – review and editing. **Philip Gribbon:** Conceptualization; supervision; validation; investigation; visualization; methodology; writing – original draft; writing – review and editing.

## Disclosure and competing interests statement

The authors declare that they have no conflict of interest.

## Supporting information



Appendix

## Data Availability

CC‐BY licence—Data contact: RD. Radical collaboration resources for this paper (PDF) are supplementary material and rolling notes will be available on Zenodo DOI: 10.5281/zenodo.10069913.

## Appendix 1 Affiliations and other resources

### Author affiliations

Dr Romain David (0000‐0003‐4073‐7456), European Research Infrastructure on Highly Pathogenic Agents (ERINHA AISBL), Brussels, Belgium, romain.david@erinha.eu.

Dr Arina Rybina (0000‐0002‐5609‐5710), Euro‐BioImaging ERIC Bio‐Hub, European Molecular Biology Laboratory (EMBL) Heidelberg, Meyerhofstrasse 1, 69117 Heidelberg, Germany, arina.rybina@eurobioimaging.eu.

Dr Jean‐Marie Burel (0000‐0002‐1789‐1861), University of Dundee, Dundee, UK, j.burel@dundee.ac.uk.

Dr Jean‐Karim Hériché (0000‐0001‐6867‐9425), European Molecular Biology Laboratory: Heidelberg, Baden‐Württemberg, Germany, heriche@embl.de.

Dr Pauline Audergon (0000‐0002‐1624‐7388), Instruct‐ERIC, Oxford House, Parkway Court, John Smith Drive, Oxford, OX4 2JY, UK, pauline@instruct-eric.org.

Dr Jan‐Willem Boiten (0000‐0003‐0327‐638X), European Advanced Translational Research Infrastructure (EATRIS)/Lygature, 3521 AL, Utrecht, The Netherlands, janwillem.boiten@lygature.org.

Dr Frederik Coppens (0000‐0001‐6565‐5145), VIB Data Core, VIB Technologies, VIB, Ghent, Belgium, frederik.coppens@vib.be.

Dr Sara Crockett (0009‐0008‐7983‐0360), BBMRI‐ERIC, Biobanking and BioMolecular Resources Research Infrastructure (BBMRI‐ERIC), Neue Stiftung Talstrasse 2, 8010 Graz, Austria, sara.crockett@bbmri-eric.eu.

Dr Katrina Exter (0000‐0002‐5911‐1536), Flanders Marine Institute, Ostend, Belgium, katrina.exter@vliz.be.

Dr Sven Fahrener (0000‐0001‐6240‐9678), IBG‐2: Plant Sciences, Forschungszentrum Jülich, 52428 Jülich, Germany, s.fahrner@fz-juelich.de

Dr Maddalena Fratelli (0000‐0002‐1769‐3427), Istituto di Ricerche Farmacologiche Mario Negri IRCCS, Milano, Italy, maddalena.fratelli@marionegri.it.

Prof Carole Goble (0000‐0003‐1219‐2137), The University of Manchester, Oxford Road, Manchester, M13 9PL, UK, carole.goble@manchester.ac.uk.

Mr Philipp Gormanns (0000‐0001‐9823‐1621), INFRAFRONTIER, Ingolstädter Landstraße 1, 85764 Oberschleißheim, Germany, philipp.gormanns@infrafrontier.eu.

Mr Tobias Grantner (0000‐0001‐5104‐5004), TU Wien, Research Unit Data Science, Vienna, Austria, tobias.grantner@tuwien.ac.at.

Dr Björn Grüning (0000‐0002‐3079‐6586), Albert‐Ludwigs‐Universität Freiburg: Freiburg, Baden‐Württemberg, Germany, bjoern.gruening@gmail.com.

Ms Kim Tamara Gurwitz (0000‐0003‐1992‐5073), European Bioinformatics Institute (EMBL‐EBI), European Molecular Biology Laboratory, Wellcome Trust Genome Campus, Hinxton, Cambridge CB10 1SD, UK, kgurwitz@ebi.ac.uk.

Prof John M. Hancock (0000‐0003‐2991‐2217), Faculty of Medicine, University of Ljubljana, Zaloška 4, 1000 Ljubljana, Slovenia, jmhancock.science@gmail.com.

Dr Henriette Harmse (0000‐0001‐7251‐9504), European Bioinformatics Institute (EMBL‐EBI), European Molecular Biology Laboratory, Wellcome Trust Genome Campus, Hinxton, Cambridge CB10 1SD, UK, henriette007@ebi.ac.uk.

Dr Petr Holub (0000‐0002‐5358‐616X), BBMRI‐ERIC, Biobanking and BioMolecular Resources Research Infrastructure (BBMRI‐ERIC), Neue Stiftingtalstrasse 2, 8010 Graz, Austria, petr.holub@bbmri-eric.eu.

Dr Nick Juty (0000‐0002‐2036‐8350), Department of Computer Science, The University of Manchester, Manchester, M13 9PL UK, nick.juty@manchester.ac.uk.

Mr Geoffrey Karnbach (0009‐0005‐9892‐7799), TU Wien, Research Unit Data Science, Vienna, Austria, geoffrey.karnbach@tuwien.ac.at.

Dr Emma Karoune (0000‐0002‐6576‐6053), Investigative Science Team, Historic England, Fort Cumberland Road, Portsmouth, P04 9LD, UK, emma.karoune@historicengland.org.uk.

Dr Antje Keppler (0000‐0003‐4358‐2269), Euro‐BioImaging ERIC Bio‐Hub, European Molecular Biology Laboratory (EMBL) Heidelberg, Meyerhofstrasse 1, 69117 Heidelberg, Germany, antje.keppler@eurobioimaging.eu.

Ms Jessica Klemeier (0009‐0006‐9083‐3808), European Molecular Biology Laboratory: Heidelberg, Baden‐Württemberg, Germany, jessica.klemeier@embl.de.

Dr Carla Lancelotti (0000‐0003‐1099‐7329), Department of Humanities, CASEs Research Group and ICREA, Universitat Pompeu Fabra, Barcelona, Spain, carla.lancelotti@upf.edu.

Dr Jean‐Luc Legras (0000‐0002‐4006‐4389), Université de Montpellier, INRAE, Institut Agro Montpellier, Montpellier, France, Jean-luc.legras@inrae.fr.

Dr Allyson L. Lister (0000‐0002‐7702‐4495), Oxford e‐Research Centre, Department of Engineering Science, University of Oxford, 7 Keble Road, OX13QG, Oxford, UK, allyson.lister@oerc.ox.ac.uk.

Dr Dario Livio Longo (0000‐0002‐6906‐9925), Istituto di Biostrutture e Bioimmagini, Consiglio Nazionale delle Ricerche, Via Nizza 52, Torino, Italy, dariolivio.longo@cnr.it.

Dr Rebecca Ludwig (0000‐0001‐9877‐3287), EATRIS ERIC, European Infrastructure for Translational Medicine, Amsterdam, 1081 HZ, The Netherlands, rebeccaludwig@eatris.eu.

Dr Bénédicte Madon (0000‐0001‐8608‐3895), Escuela Superior de Ingenieros, Universidad de Sevilla/AICIA‐Asoc.Invest. Coop.Ind. Andalucía, Camino de los Descubrimientos s/n, Sevilla‐41092, Spain, bcg.madon@gmail.com.

Dr Marzia Massimi (0000‐0001‐5052‐1822), Istituto di Biochimica e Biologia Cellulare, Consiglio Nazionale delle Ricerche (CNR), I‐00015 Monterotondo, Rome, Italy, marzia.massimi@cnr.it.

Dr Vera Matser (0000‐0002‐2010‐6844), The Alan Turing Institute, British Library, 96 Euston Road, London, NW1 2DB, UK, vmatser@turing.ac.uk.

Dr Rafaele Matteoni (0000‐0002‐0314‐5948), Istituto di Biochimica e Biologia Cellulare, Consiglio Nazionale delle Ricerche (CNR), I‐00015 Monterotondo, Rome, Italy, rafaele.matteoni@cnr.it.

Dr Michaela Th Mayrhofer (0000‐0001‐6932‐0473), Biobanking and Biomolecular Resources Research Infrastructure (BBMRI‐ERIC), 8010, Graz, Austria, michaela.th.mayrhofer@bbmri-eric.eu.

Prof Christian Ohmann (0000‐0002‐5919‐1003), European Clinical Research Infrastructure Network (ECRIN), Kaiserswerther Str. 70, 40477 Düsseldorf, Germany, christianohmann@outlook.de.

Dr Maria Panagiotopoulou (0000‐0002‐4221‐7254), European Clinical Research Infrastructure Network (ECRIN), 5‐7 rue Watt 75013 Paris, France, maria.panagiotopoulou@ecrin.org.

Dr Helen Parkinson (0000‐0003‐3035‐4195), European Bioinformatics Institute (EMBL‐EBI), European Molecular Biology Laboratory, Wellcome Trust Genome Campus, Hinxton, Cambridge CB10 1SD, UK, parkinso@ebi.ac.uk.

Dr Isabelle Perseil (0000‐0001‐9058‐9290), INSERM, 101 rue de Tolbiac, 75013 Paris, France, isabelle.perseil@inserm.fr.

Dr Claudia Pfander (0000‐0002‐9574‐9553), Euro‐BioImaging ERIC Bio‐Hub, European Molecular Biology Laboratory (EMBL) Heidelberg, Meyerhofstrasse 1, 69117 Heidelberg, Germany, claudia.pfander@eurobioimaging.eu.

Dr Roland Pieruschka (0000‐0002‐9774‐2670), IBG‐2: Plant Sciences, Forschungszentrum Jülich, 52428 Jülich, Germany, r.pieruschka@fz-juelich.de.

Dr Michael Raess (0000‐0002‐8759‐1186), INFRAFRONTIER GmbH: Neuherberg/München, Germany, michael.raess@infrafrontier.eu.

Prof Andreas Rauber (0000‐0002‐9272‐6225), TU Wien, Research Unit Data Science, Vienna, Austria, andreas.rauber@tuwien.ac.at.

Dr Audrey S. Richard (0000‐0002‐0207‐0139), European Research Infrastructure on Highly Pathogenic Agents (ERINHA), 98 rue du Trône B‐1050 Brussels, Belgium, audrey.richard@erinha.eu.

Dr Paolo Romano (0000‐0003‐4694‐3883), IRCCS Ospedale Policlinico San Martino, Largo Rosanna Benzi 10, 16132 Genova, Italy, paolo.romano@hsanmartino.it.

Dr Antonio Rosato (0000‐0001‐6172‐0368), Department of Chemistry and Magnetic Resonance Center, University of Florence: Sesto Fiorentino, Italy, antonio.rosato@unifi.it.

Dr Alex Sánchez‐Pla (0000‐0002‐8673‐7737), Universitat de Barcelona, Vall d'Hebron Institut de Recerca, Barcelona, Spain, alex.sanchez@vhir.org.

Prof Susanna‐Assunta Sansone (0000‐0001‐5306‐5690), Oxford e‐Research Centre, Department of Engineering Science, University of Oxford, 7 Keble Road, OX13QG, Oxford, UK, sa.sansone@gmail.com.

Dr Ugis Sarkans (0000‐0001‐9227‐8488), European Bioinformatics Institute (EMBL‐EBI), European Molecular Biology Laboratory, Wellcome Genome Campus, Hinxton, Cambridge CB10 1SD, UK, ugis@ebi.ac.uk.

Dr Beatriz Serrano‐Solano (0000‐0002‐5862‐6132), Euro‐BioImaging ERIC Bio‐Hub, European Molecular Biology Laboratory (EMBL) Heidelberg, Meyerhofstrasse 1, 69117 Heidelberg, Germany, beatriz.serrano.solano@eurobioimaging.eu.

Dr Jing Tang (0000‐0001‐7480‐7710), University of Helsinki, Helsinki, Finland, jing.tang@helsinki.fi.

Dr Ziaurrehman Tanoli (0000‐0003‐2435‐9862), Institute for Molecular Medicine Finland, University of Helsinki, Helsinki, Finland, zia.rehman@helsinki.fi.

Dr Jonathan Tedds (0000‐0003‐2829‐4584), ELIXIR Hub, Wellcome Genome Campus, Hinxton, CB10 1SD, UK, jonathan.tedds@elixir-europe.org.

Mr Harald Wagener (0000‐0003‐1073‐4991), Group Leader Cloud and HPX, Center for Digital Health, BIH@Charité University Medicine Berlin, Berlin, Germany, harald.wagener@charite.de.

Mr Martin Weise (0000‐0003‐4216‐302X), TU Wien, Research Unit Data Science, Vienna, Austria, martin.weise@tuwien.ac.at.

Mr Hans V. Westerhoff (0000‐0002‐0443‐6114), University of Amsterdam, Free University Amsterdam, University of Manchester, NL, h.v.westerhoff@uva.nl.

Mr Rudolf Wittner (0000‐0002‐0003‐2024), BBMRI‐ERIC, Biobanking and BioMolecular Resources Research Infrastructure (BBMRI‐ERIC), Neue Stiftingtalstrasse 2, 8010 Graz, Austria, rudolf.wittner@bbmri-eric.eu.

Dr Jonathan Ewbank (0000‐0002‐1257‐6862), European Research Infrastructure on Highly Pathogenic Agents (ERINHA), 98 rue du Trône B‐1050 Brussels, Belgium, jonathan.ewbank@erinha.eu.

Dr Niklas Blomberg (0000‐0003‐4155‐5910), ELIXIR Hub, Wellcome Genome Campus, Hinxton, CB10 1SD, UK, niklas.blomberg@elixir-europe.org.

Dr Philip Gribbon (0000‐0001‐7655‐2459), ITMP Head Discovery Research (ITMP Drug Discovery), Germany philip.gribbon@itmp.fraunhofer.de.

### Online *EOSC* and *EOSC‐Life* resources cited **[R2]**

Blomberg N, Sarntivijai S, Mayrhofer MTh (2020) EOSC‐Life Data Management Plan, Zenodo https://doi.org/10.5281/zenodo.4010442

Boiten JW, Ohmann C, Adeniran A, Canham S, Cano Abadia M, Chassang G, Chiusano ML, David R, Fratelli M, Gribbon P (2021a) EOSC‐LIFE WP4 TOOLBOX: Toolbox for sharing of sensitive data – a concept description. https://doi.org/10.5281/zenodo.4483694 [PREPRINT]

Boiten JW, Ohmann C, Adeniran A, Canham S, Cano Abadia M, Chassang G, Chiusano ML, David R, Fratelli M, Gribbon P (2021) EOSC‐Life Guidance and policy on standards and tools to facilitate sharing and reuse of multimodal data (including imaging), cohort integration, and biosamples. https://doi.org/10.5281/zenodo.4591011

ELIXIR (2021) Research Data Management Kit. A deliverable from the EU‐funded ELIXIR‐CONVERGE project (grant agreement 871075). https://rdmkit.elixir‐europe.org

EMDB (the Electron Microscopy Data Bank), a public repository for electron cryo‐microscopy maps and tomograms of macromolecular complexes and subcellular structures. https://www.ebi.ac.uk/emdb/

EOSC TF sustainability https://www.eoscsecretariat.eu/working‐groups/sustainability‐working‐group

Goble C, Soiland‐Reyes S, Bacall F, Owen S, Pireddu L, Leo S (2023) EOSC‐Life Implementation of a mechanism for publishing and sharing workflows across instances of the environment. https://doi.org/10.5281/zenodo.7886545

Goble C, Bacall F, Soiland‐Reyes S, Owen S, Williams A, Eguinoa I, Droesbeke B, Ménager H, Rodríguez Navas L, Fernández JM *et al* (2022) WorkflowHub – a FAIR registry for workflows. *F1000Research*, 11 https://doi.org/10.7490/f1000research.1118984.1

Goble C, Soiland‐Reyes S, Bacall F, Owen S, Williams A, Eguinoa I, Droesbeke B, Leo S, Pireddu L, Rodríguez‐Navas L *et al* (2021) Implementing FAIR Digital Objects in the EOSC‐life workflow collaboratory. https://doi.org/10.5281/zenodo.4605654 [PREPRINT]

Gribbon P, Parkinson H, Witt G (2020) D1.1 EOSC‐Life EOSC Cloud feasibility assessment for demonstrators. https://doi.org/10.5281/zenodo.4010371

Haley N, Leitner F, Bischof J, Audergon P (2020) EOSC‐Life Publication of generic guidelines for the organisation of topic‐specific Open Calls. https://doi.org/10.5281/zenodo.4048442

Kerfant C, Ruiz‐Pérez J, García‐Granero J, Lancelotti C, Madella M, Karoune E (2022) A dataset for assessing phytolith data for implementation of the FAIR data principles. https://doi.org/10.5281/zenodo.7215804

Lamanna G, Bird I, Petzold A, Asmi A, Brus M, Blomberg N, Räß M, Dimper R, Gotz A, Dekker R *et al* (2021) ESFRI Science clusters position statement on expectations and long‐term commitment in open science. https://doi.org/10.5281/zenodo.4892245

Jahn N, Laakso M, Lazzeri E, McQuilton P (2023) Study on the readiness of research data and literature repositories to facilitate compliance with the Open Science Horizon Europe MGA requirements (Version 1.0). https://doi.org/10.5281/zenodo.7728016

Nardello I, Asmi A, Buch R, Bos EJ (2022) Delivering for EOSC – key exploitable results of Horizon 2020 EOSC‐related Projects (FULL Report). https://doi.org/10.5281/zenodo.7401539

Parkinson H, Gribbon P, Sarkans U, Witt G, Zaliani A, Kohler M, Swedlow J, Burel JM, Swertz M, van Enckevort E *et al* (2021) EOSC‐Life EOSC repository deployment for project demonstrators. https://doi.org/10.5281/zenodo.4432029

Parkinson H, Gribbon P, Sarkans U, Witt G, Zaliani A, Kohler M, Swedlow J, Burel JM, Swertz M, van Enckevort E *et al* (2022) EOSC‐Life EOSC FAIR services deployment for open calls. https://doi.org/10.5281/zenodo.6208249

Perseil I, Sansone SA, McQuilton P, Dumontier M, Roos M, Bonino L, Wittner R, Holub P, Geiger J *et al* (2020) EOSC‐Life FAIR requirements document. https://doi.org/10.5281/zenodo.3693883

Robertson D, Meijer J, Jones B, Pérez Gómez A, Roi A, Lecarpentier D, Hacque‐Cosson F, Ilijasic Versic I, Szprot J, Hrušák J *et al* (2022) Towards sustainable funding models for the European Open Science Cloud. Financial Sustainability Task Force Progress report https://www.eosc.eu/sites/default/files/2022‐11/financial‐sustainability‐tf‐progress‐report‐nov‐2022.pdf

Romano P, Aznar R, López‐Coronado JM, Vasilenko A, Robert V, Wade G, Mineau‐Cesari J, Legras, JL (2023) Microbial resources in the European Open Science Cloud: MIRRI contribution to the EOSC‐Life project. ECCO XL 2022, Braunschweig, September 27–29, 2022. https://doi.org/10.5281/zenodo.8052705

Rybina A, Audergon P, Pfander C (2023) EOSC‐Life report on the work of the open call projects. https://doi.org/10.5281/zenodo.8263074

Wilkinson MD, Sansone SA, Grootveld M, Nordling J, Dennis R, Hecker D (2022). FAIR assessment tools: towards an “Apples to Apples” comparisons. https://doi.org/10.5281/zenodo.7463421

Zafeiropoulos H, Beracochea M, Ninidakis S, Exter K, De Moro G, Potirakis A, Richardson L, Corre E, Machado J, Pafilis E *et al* (2023). metaGOflow: a workflow for the analysis of marine Genomic Observatories shotgun metagenomics data – use case (v1.0.0) [DATASET]. https://doi.org/10.5281/zenodo.7771821

Other resources are available at: https://zenodo.org/communities/eosc‐life/

**Table:**

Organisation acronyms, names and links	URL
BBMRI: Biobanking and Biomolecular Resources Research Infrastructure	https://www.bbmri‐eric.eu/
EATRIS: European infrastructure for translational medicine (European Advanced Translational Research Infrastructure in Medicine)	https://eatris.eu/
EBI: European Bioinformatics Institute	https://www.ebi.ac.uk/
EC: European Commission	https://commission.europa.eu
ECRIN: European Clinical Research Infrastructure Network	https://ecrin.org/
ELIXIR	https://elixir‐europe.org/
EMBL: European Molecular Biology Laboratory	https://www.embl.org/
EMBRC: European Marine Biological Resource Centre	https://www.embrc.eu/
ERINHA: European Research Infrastructure on Highly Pathogenic Agents	http://www.erinha.eu
EOSC: European Open Science Cloud	https://eosc‐portal.eu/
ESFRI: European Strategy Forum Research Infrastructure	https://www.esfri.eu/
EU‐OPENSCREEN	https://www.eu‐openscreen.eu/
Euro‐BioImaging	https://www.eurobioimaging.eu/
GA4GH: Global Alliance for Genomics and Health	https://www.ga4gh.org/
GSO: Group of Senior Officials on global Research Infrastructures	http://www.gsogri.org/
INFRAFRONTIER	https://www.infrafrontier.eu/
INSTRUCT	https://instruct‐eric.org/
ISBE: Infrastructure for Systems Biology in Europe	https://cordis.europa.eu/project/id/312455
MIRRI: Microbial Resource Research Infrastructure	https://www.mirri.org/
NEUBIAS: Network of EUropean BioImage Analysts	https://eubias.org/NEUBIAS/
NHGRI: National Human Genome Research Institute	https://www.genome.gov
OECD: Organisation for Economic Co‐operation and Development	https://www.oecd.org/
RDA: Research Data Alliance	https://www.rd‐alliance.org/

Project acronyms, names, and links	URL
AgroServ	https://agroserv.eu/
AI4Life	https://ai4life.eurobioimaging.eu
BIOINDUSTRY 4.0	https://cordis.europa.eu/project/id/101094287
BY‐COVID	https://by‐covid.org/
CanSERV	https://www.canserv.eu/
CORBEL (Coordinated Research Infrastructures Building Enduring Life‐science Services)	https://www.corbel‐project.eu
DANUBIUS‐IP	https://www.danubius‐ri.eu/
EMPHASIS (Effective management of pests and harmful alien species – Integrated solutions)	https://www.emphasisproject.eu/
ENVRI‐FAIR	https://envri.eu/home‐envri‐fair/
ESCAPE	https://projectescape.eu/
EOSC4CANCER	https://eosc4cancer.eu/
EOSC‐Life	https://www.eosc‐life.eu/
EuroScienceGateway	https://galaxyproject.org/projects/esg/
FAIRplus	https://fairplus‐project.eu/
FAIR Phytolith project	https://open‐phytoliths.github.io/FAIR‐phytoliths/
FAIRsharing	https://fairsharing.org/
ISIDORe (Integrated Services for Infectious Disease Outbreak Research)	https://isidore‐project.eu/
PaNOSC	https://www.panosc.eu/
Precision Toxicology	https://cordis.europa.eu/project/id/965406
SSHOC	https://sshopencloud.eu/

Other links in footnotes – Part I	URL
ABS Permits	https://oneworldanalytics.com/abspermits/
Amsterdam Declaration on Funding Research Software Sustainability Draft	https://future‐of‐research‐software.org/draft‐amsterdam‐declaration‐on‐funding‐research‐software‐sustainability/
BioContainers	https://biocontainers.pro/
Biological systems modelling JWS Online	https://jjj.biochem.sun.ac.za/
Biomodels	https://www.ebi.ac.uk/biomodels/
Biotracks	https://github.com/CellMigStandOrg/biotracks
Codebases General Guide For Exploring Large Open Source Codebases	https://pncnmnp.github.io/blogs/oss‐guide.html
Conda	https://docs.conda.io/en/latest/
Convention on Biological Diversity (CBD) – Recent agreements	https://www.cbd.int/doc/c/e6d3/cd1d/daf663719a03902a9b116c34/cop‐15‐l‐25‐en.pdf
COVID‐19 data portal	https://www.covid19dataportal.org/
DockerHub	https://hub.docker.com/
ELIXIR's Long‐term sustainability plan (2019)	https://f1000research.com/documents/8‐1642
EMO BON	https://www.embrc.eu/emo‐bon
EOSC‐Life Calls	https://www.eosc‐life.eu/calls/
EOSC‐Life courses websites	https://www.eosc‐life.eu/news/training‐modelling‐covid‐19‐epidemics/
EOSC‐Life Internal Call for Academia‐Industry Collaborations	https://www.eosc‐life.eu/industrycall/
EOSC‐Life Training Open Call	https://www.eosc‐life.eu/services/open‐call‐training/
EOSC‐Life Training	https://www.eosc‐life.eu/services/training/
EOSC SRIA MAR (Strategic Research & Innovation Agenda and its Multi‐Annual Roadmap)	https://www.eosc.eu/sria‐mar
EOSC Task Forces	https://eosc.eu/eosc‐task‐forces
EOSC Task Force PIDs policy and Implementation	https://www.eosc.eu/advisory‐groups/pid‐policy‐implementation
FAIR Cookbook	https://faircookbook.elixir‐europe.org
FAIRsharing Community champions program	https://fairsharing.org/community_champions
FAIRsharing educational material	https://fairsharing.org/educational
Fragalysis	https://fragalysis.diamond.ac.uk/viewer/react/landing/
GDPR	https://gdpr‐info.eu/
GitHub	https://github.com/
GWAS studies	https://www.ebi.ac.uk/gwas/docs/methods/summary‐statistics
INFRASERV	https://rea.ec.europa.eu/funding‐and‐grants/horizon‐europe‐research‐infrastructures/research‐infrastructure‐services‐support‐health‐research‐accelerate‐green‐and‐digital‐transformation_en
ISA Tools	https://isa‐tools.org
ISO 23494	https://www.iso.org/standard/80715.html

Other links in footnotes – Part II	URL
Life‐Monitor	https://app.lifemonitor.eu/
Linked Data	https://www.w3.org/DesignIssues/LinkedData
Long‐Term Sustainability of Research Infrastructures (ESFRI Report)	https://www.esfri.eu/sites/default/files/u4/ESFRI_SCRIPTA_VOL2_web.pdf
Memorandum of Understanding (MoU) with EuroBioimaging and Instruct‐ERIC	https://www.eu‐openscreen.eu/newsroom/eu‐openscreen‐news/ansicht/eu‐openscreen‐has‐signed‐a‐memorandum‐of‐understanding‐mou‐with‐eurobioimaging‐and‐instruct‐eric.html
MGnify	https://docs.mgnify.org/src/docs/analysis.html
MIAPPE	https://www.miappe.org/
Microscopy data analysis	https://www.ebi.ac.uk/training/events/microscopy‐data‐analysis‐0/
Microscopy data analysis: Machine Learning and Bioimage archive	https://www.ebi.ac.uk/training/events/microscopy‐data‐analysis‐machine‐learning‐and‐bioimage‐archive‐2022/
Nagoya Protocol on Access and Benefit‐sharing	https://www.cbd.int/abs/
NAR	https://www.oxfordjournals.org/nar/database/c/
NEUBIAS Training school	https://www.eosc‐life.eu/news/neubias‐defragmentation‐training‐school/
Ontology Cross Reference Service (OxO)	https://www.ebi.ac.uk/spot/oxo/
Ontology Lookup Service (OLS)	https://www.ebi.ac.uk/ols4
Open Microscopy Environment Next‐Generation File Formats (OME‐NGFF)	https://ngff.openmicroscopy.org
OSSDIP	https://www.ifs.tuwien.ac.at/infrastructures/ossdip/
Paper Sprint Method (Michigan University)	https://sph.umich.edu/cehr/pdf/Paper_Sprint_Manual.pdf
Permissive Licences	https://fossa.com/blog/all‐about‐permissive‐licences/
Quay.io	https://quay.io/
RDMKit	https://rdmkit.elixir‐europe.org/
Recommendation of the Council on Enhancing Access to and Sharing of Data	https://legalinstruments.oecd.org/en/instruments/OECD‐LEGAL‐0463
Sourceforge	https://sourceforge.net/
TravisCI	https://www.travis‐ci.com/
World‐cafe	https://theworldcafe.com/key‐concepts‐resources/world‐cafe‐method/
WorkflowHub	https://workflowhub.eu/
