# Supplementary material for: “Be sustainable”: EOSC‐Life recommendations for implementation of FAIR principles in life science data handling
Source: EMBO J. 2023 Nov 15;42(23):e115008. doi: 10.15252/embj.2023115008 (PMC10690449; doi:10.15252/embj.2023115008)
Supplement: Supplementary file 1 — Appendix [file EMBJ-42-e115008-s001.docx]

Appendix 2: supplementary information

**Supplementary_Information_S1 Radical collaboration**® **method for analysis of sustainability strategies employed in EOSC-Life and identification of best practice, including preparation of this manuscript**

We have applied a set of theories and concepts, termed radical collaboration, to identify key elements that support the sustainability of the work and results produced within the framework of our inter-RI project. The choice of taking a radical collaboration approach was made because it intrinsically supports processes involving skilled people working in a collaborative environment. The methodology of radical collaboration was initially established for the purposes of conflict resolution and mediation (Tamm & Luyet, 2010). Further extending the range of application McGovern (2018a, p.6) states, “The concept of radical collaboration means coming together across disparate, but engaged, domains in ways that are often unfamiliar or possibly uncomfortable to member organisations and individuals to identify and solve problems together, to achieve more together than we could separately.” The reuse of radical collaboration as an operational framework in research was also suggested by McGovern (2018b) in a second contribution in that issue of *Research Library Issues*, based on an earlier proposition by Scott (2017 & 2019).

In 2018, Ruttenberg and Waraksa brought out a special issue of *Research Library Issues* that placed a special emphasis on the development of a collaborative thesis for RDM communities. In parallel, Nurnberger (2018) described how the radical collaboration methodology was applied to the experience of forming and maintaining Research Data Alliance (RDA) groups, offering specific recommendations for developing sustainable institutional and inter-RIs RDM services.

When considering issues related to the sustainability of RDM, it is important to recognise that these matters and the underlying research are strongly influenced by the temporary nature of research projects and associated funding. These issues have been put into focus by the publication of the FAIR principles (Wilkinson et al., 2016), which provided a framework for driving reuse of data and software resources. Achieving reuse of scientific products of large collaborative projects, involving many partners, has led to the creation of vital forums and international assemblies, such as the Research Data Alliance (RDA), GO-FAIR initiative, European Open Science Cloud (EOSC), World Data System (WDS), and Committee on Data (CODATA) of the International Science Council (ISC). These forums and assemblies typically aim to both invest in new resources as well as increase the sustainability of outcomes and the reuse of the deliverables and tools resulting from inter-organisation projects.

In her first paper, McGovern (2018a) developed three working concepts that can be used “*to guide the process of applying the radical collaboration methodology: inclusive community, distributed digital practices, and productive and sustainable collaboration*” **[R11]**. We used this theoretical framework in our study to assess the durability and sustainability of EOSC-Life outputs. We subsequently applied the methodology shown by Pickering et al, in 2020 in the development of the present paper. Specifically, we asked the project participants to share their perceptions about the sustainability of the work they carried out on their tasks in specifically focused meetings run as sprint events. The first steps were about developing a detailed plan with willingness to make inputs. The team organised the first meeting to present the focus of the paper, define methodology, and recruit skilled people with a good representation of the different EOSC-Life working teams and RIs. 13 meetings were organised then we run 6 Paper Sprints with iterative invitations through EOSC-Life community for a total of 28 open meetings (integrating also EOSC-Life partners recruited with call for projects).

Jake Knapp, a Google employee presented in 2016 sprints as a method to enable project teams to develop and prototype new products rapidly. These sprints were later adapted to apply the approach in academia, and a helpful paper sprint manual was developed by the University of Michigan School of Public Health Center (Sinha et al., 2018). The paper sprint method^^[[1]](#footnote-1)^^ was ideal for our purpose, as it enabled sections of the research paper to be written mutually and simultaneously by multiple members of a research team. Such a similar collaborative method is also applied to generate content, at a good rate, for RDMkit.

In the first paper sprint, the team wrote the case studies parts with the participants’ perspectives, identified relevant examples for sustainability, and planned the next sprint steps. In the 2 secondary sprint(s), we met online to write the remaining portions of the paper based on the results of meetings and exchanges. The final sprints were performed to reorganise the paper and to shorten longer parts.

We focussed on gathering the participants’ perspectives on the efficacy of the capacity building process, such as identifying training needs and carrying out the open call process. The capacity building process was implemented to engage and support the development and extension of FAIR resources and services both inside and outside of the EOSC-Life consortium. One point of particular interest was examining the participants’ perceptions of how FAIR components were integrated in projects funded by EOSC-Life involving the 13 RIs^^[[2]](#footnote-2)^^,^^[[3]](#footnote-3)^^,^^[[4]](#footnote-4)^^ how these projects were managed, and how sustainable the project outcomes were. We summarised the FAIR components, including reusable elements of the research data management plan (DMP), Authentication and Authorisation Infrastructures services (AAIs), data repositories, data feeds i.e. how data is served in data federations, and data access governance e.g. sensitive data aspects. This summary was prepared by using community-approved terminology and by referring to provenance information. Once the paper sprint process completed, the entire manuscript was subjected to a thorough and iterative review by the involved authors.

**Supplementary_Information_S2 Collaborative User projects**

The first tranche involved a set of seven demonstrators drawn from the RIs community, placing a focus on achieving sustainable, interoperable collaboration between two or more research infrastructures. The underlying aims were to share experience between resources, to make data more interoperable, and to evaluate sustainability strategies, development of shared processes, improving portability of data, analysis and resources and code.

Subsequent open calls involved three sub-call topics: i) digital LSs (broad application), ii) sensitive data, and iii) academic-industry collaboration. The aim was to support projects that would populate EOSC with FAIR LS data, cloud-based tools, resources, and workflows from a broad range of LS domains.

To support the long-term sustainability of EOSC, all stakeholders in the scientific community including SMEs and industry have to be engaged, realise the potential of/benefit from the collaborative cloud ecosystem. EOSC-Life therefore explored ways of collaboration between public and commercial entities for the development and integration of cloud-based solutions. After consultation with industry representatives on an appropriate framework for applications and projects, EOSC-Life launched a dedicated Internal Call for Academia-Industry^^[[5]](#footnote-5)^^ collaborations that resulted in support for two feasible projects. These projects involved consultations with EOSC-Life experts as well as the implementation of EOSC-Life resources.

In the digital LSs call, a new online platform, fragalysis^^[[6]](#footnote-6)^^, was developed enabling rapid access to data from fragment screens in a collaborative environment. This innovative platform had a major impact in COVID-19 related work by allowing fast release of fragment screens to the worldwide community. From a community aspect, the FAIR Phytoliths^^[[7]](#footnote-7)^^ project developed a new ontology and a set of domain-specific FAIR guidelines for a community which has historically poor data sharing practices (Kerfant et al., 2022). Such efficient cross-discipline collaboration beyond the traditional ESFRI clusters has been driven and supported by integrating projects in EOSC-Life that extend into other sectors.

The project *"Integrating several EU-RI datasets with focus on preclinical and discovery research bioimaging"* (Ref PID 14176) demonstrates a collaborative effort of three RIs in the field of biomedical imaging (Euro-BioImaging), animal model strains (INFRAFRONTIER^^[[8]](#footnote-8)^^), and biological chemistry (EU-OPENSCREEN). The three RIs joined forces and combined their resources by building novel, open-source tools for integrating and aligning information associated with data belonging to their infrastructures. Another set of examples from open call projects originated from collaborative work among the RIs Euro-BioImaging, ELIXIR, and ISBE. These projects have led to the more thorough integration of data from imaging, the scientific literature, and modelling, and have provided (and exemplified) more validated mathematical models as live data repositories for mammalian cell biology and ageing (Kolodkin et al., 2020).

To highlight the sustainability aspects faced by the various open call projects, we take a closer look at one project, MetaGOflow, first as one the seven demonstrators then as an Open Call. MetaGOflow (Zafeiropoulos et al., 2023), a workflow for marine genomics observatory data analysis, was built by a team from EMBRC to analyse the eDNA data from its EMO BON^^[[9]](#footnote-9)^^ projects that are used to study European marine biodiversity. MetaGOflow is based on an existing platform, MGnify^^[[10]](#footnote-10)^^, and provides taxonomic inventories and functional analyses of use to LS researchers in the field of marine biology. It can also be used by researchers in other fields of biology. MetaGOflow promoted sustainability through continued re-use by enhancing the efforts of EMO BON: the eDNA data collected by EMO BON are now turned into scientific results that can be used for academic research and for environmental reporting. The data (raw and analysed) become more interesting to a wider audience and are more frequently used, increasing the chance that they will continue to be provided (organisational and financial sustainability). As the workflow produces full provenance for all its inputs and outputs, the data are more likely to be (i) trusted and (ii) re-used, again promoting its sustainability.

**Supplementary_Information_S3 The COVID-19 data portal**

During the COVID-19 pandemic, the importance of sustaining tools, services, best practices, and guidelines for sensitive data management was clearly demonstrated. In a crisis of this nature, rapid access to accurate data can enable researchers to assess the severity, spread, and impact of a pandemic and result in the implementation of efficient, effective response strategies. In their recommendations for tackling COVID-19, the Research Data Alliance (RDA) clearly states that data should be deposited in repositories to facilitate quality control, timely sharing, and sustainable access. Whenever possible, these should be trustworthy data repositories that have been certified, are subject to rigorous governance, and are committed to promoting the long-term sustainability of their data holdings (RDA, 2020).

The COVID-19 data portal established in EOSC-Life, contains over 16 million raw and assembled sequences and analysis of SARS-CoV-2 and other coronaviruses. FAIR interoperability standards represent key elements that ensure the sustainability of these resources. Due to the urgent need met by the resources, mid-term sustainability has been achieved through the follow-up funding of the BY-COVID project.

**Supplementary_Information_S4 The FAIR Cookbook as a resource to support sustainable FAIR implementation**

The FAIR Cookbook (Rocca-Serra et al, 2023) was created and is managed by professionals who routinely work with data in academia, (bio)pharmaceutical companies and information service industries. It is an open and collaborative resource, which documents the fundamental processes and capabilities for provisioning FAIR data and services, and provides real examples and use cases by data-producing projects and organisations. Recommended by Horizon Europe, and anchored to several ELIXIR Nodes for long term sustainability, the FAIR Cookbook serves as practical guidance to improve every-day tasks, and contributes to a curriculum on FAIR data, informing discussions around the necessary changes to deliver FAIR within organisations. The success of the FAIR Cookbook has been to cultivate the collective knowledge, in academia and industry, to timely deliver specialised content, which fills the glaring gap between high-level FAIR Principles and their actual implementation in the LSs.

**Supplementary_Information_S5 Ontology services extended and supported by EOSC-Life**

The Ontology Lookup Service (OLS) is an ontology discovery and access service containing to date 242 biomedical ontologies, 7,790,912 terms, 42,514 properties, and 22,653 individuals. OLS features a web interface that scientists can use to search for and visualise ontologies as well as a highly accessed API. EOSC-Life has resulted in over 35 additional ontologies being made available in OLS, representing user needs. Ontologies are continually updated, and new ontologies are being added once they mature to the point that they meet users’ needs. The aligned resource, the Ontology Cross Reference Service (OxO), provides cross-ontology mapping service allowing users to integrate datasets that are annotated with different ontology terms, a common use case when working data integration across datasets, projects, or biomedical domains. Ontologies in OLS are used to deliver cross references in OxO offering a convenient way for users to access cross references. Zooma is an ontology used for metadata mapping applications. This allows ontologies to be applied to text by users such as biomedical curators and data annotators.

**Supplementary_Information_S6 RO-Crate and LifeMonitor, EOSC-Life tools for sustainable workflows**

RO-Crate allows researchers to package and aggregate research artefacts with their metadata and relationships, as well as readily package and exchange workflows, including the needed information regarding provenance. RO-Crate was used in the context of the case study MetaGOFlow. The workflow outputs are, in that study, shared through MGnify and additionally packaged as RO-Crate on the EMO BON Github repository: the data inputs, data outputs, and the full provenance (meta)data. Sustainability through continued re-use as well as organisational sustainability are guaranteed for the foreseeable future.

Building on this, EOSC-Life developed the LifeMonitor, a service that facilitates the maintenance of computational processes and supports their reusability over time through periodic testing and test monitoring. Monitored workflows are less likely to break down over time and consequently are more sustainable. The workflows are registered in WorkflowHub.

**Supplementary_Information_S7 Case study on issues related to compliance when gathering digital sequence data**

One case study involves the Nagoya Protocol on Access and Benefit-sharing. To gather biological samples in the field to obtain their genetic material, scientists are required to comply with the ABS requirements (where, what, and why they are collecting). They must negotiate the terms by which any benefits that might arise from utilising the genetic material will be shared. The physical samples and derived results must be linked to these permits. At present, the ABS requirements are limited to physical samples: digital sequence information (DSI) are not in scope and so ABS permits^^[[11]](#footnote-11)^^ are not relevant for the sustained use of archived sequences derived from such sampled material. However, discussions to extend the scope to include DSI have been ongoing, and the outcomes of those will have an impact on how scientists and archives deal with allowing the DSI data to be (legally) sustainably accessible and re-usable. Recent agreements^^[[12]](#footnote-12)^^ to develop a multilateral system for benefit sharing from DSI, will increase the burden on the sharing of DSI while at least still recognising the benefits of such DSI for biodiversity conservation. Given the significant impact the convention’s decisions will have on the sustainable archiving and sharing of DSI, all parties including scientists, biobanks, and data portals will do their best to link their digital data to the ABS permits of the genetic resources from where those data were obtained. This will allow for sustainability by allowing for (legally) correct data re-use.

**Supplementary_Information_S8 Sensitive data toolbox**

The toolbox provides links to recommendations, procedures, and best practices, as well as to software tools that support data sharing and reuse. The concept of the toolbox has been submitted to Zenodo (Boiten et al., 2021a & 2021b) and subsequently a prototype has been implemented with a dedicated tagging system. Both the evaluation of the initial tagging system (Ohmann et al., 2022) and the development of the full version (David et al., 2022) have been published.

A considerable degree of dissemination was achieved by the publication and it has been downloaded more than 3000 times, in the 98th percentile of the 435,336 tracked articles of a similar age in all journals. Nevertheless, the toolbox will only become a useful resource for biomedical researchers when the sustainability beyond the project’s lifetime can be assured. Two high-level models for sustainability are being evaluated: a) an organisation takes full responsibility (model for the EBI databases), or b) a community takes responsibility, either funded by future grants or through “in kind” contributions from intrinsically motivated research parties/individuals (as is happening in various open-source communities). In either model, not only the technical and operational support of the toolbox should be sustained, but also an editorial board securing the actuality and correctness of the content.

While the joint RI communities in EOSC-Life clearly showed interest in using and maintaining the toolbox, the lack of clarity on its long-term sustainability leads to a reluctance to seriously communicate and disseminate it among the user communities **[R2]**. Similarly, RIs tend to be reluctant to augment the contents of the toolbox to the level required to serve as an authoritative resource. To avoid this catch-22, a group of novice users (i.e. users not exposed to the toolbox before) recruited across the participating RIs will test the toolbox and propose missing content. Any content deemed broadly usable will be added by the editorial board before the end of the project.

**Supplementary_Information_S9 The FAIRsharing catalogue**

FAIRsharing promotes the FAIR Principles by promoting the value and use of data and metadata standards, and their use by databases. For this reason, the FAIRsharing API serves a growing number of tools and services that use it for look-up, selection and content retrieval of standards and repositories in the: (i) creation of data management plans, (ii) enrichment of guidance and training material, and (ii) assessment of and assistance with FAIRness, work in progress under the EOSC FAIR Metrics and Data Quality Task Force (Wilkinson et al, 2022). Cultivating the collective knowledge, engaging the community, and becoming a core element of the ecosystem are the key essentials for the success and long-term sustainability of any resources. To address this challenge, FAIRsharing has also launched the Community Champion Programme^^[[13]](#footnote-13)^^, a thriving community of domain and discipline experts, which also includes members of EOSC-Life, who: (i) act as advocates to promote the value of standards, databases and policies for digital objects (incl. data, software); (ii) create educational material^^[[14]](#footnote-14)^^ describing these resources helping researchers and other stakeholders to find, use and adopt them, and (iii) enrich the content of FAIRsharing, adding and enhancing the description and discoverability of these resources.

**Supplementary_Information_S10 Implementation of Open call training programs to build sustainability**

Development of reusable training resources to support sustainability of EOSC-Life project outcomes has been one of the high priorities for Open and Internal Calls projects. A step-by-step training and procedures for setting up the framework to build and manage an ontology was produced while developing the first Phytolith ontology with the help of EOSC-Life experts. The materials could be used as orienting and guiding help for other communities aiming to create ontology for their scientific area. Within this collaborative training framework a series of Open Research skills workshops was run through support by EOSC-Life Training Open Calls. Specifically, EOSC-Life experts were brought together to co-create two workshops on standard vocabularies, ontologies and FAIR data. These efforts enabled the project to go above and beyond the goals initially set by the project and to deliver an ontology using sustainable and portable processes used by the community. (Publication in preparation).

**Supplementary_Information_S11 Applicability of the EOSC-Life lessons learnt for the sustainability of the ISIDORe project and other connected communities**

ISIDORe (Integrated Services for Infectious Disease Outbreak Research) is the broadest of the current INFRASERV programmes. Led by the European Research Infrastructure on Highly Pathogenic Agents (ERINHA), it brings together 17 research infrastructure and networks, providing services covering all experimental approaches, and also including regulatory advice and the social sciences. The services are offered under the transnational access model, with funding being provided to the research infrastructure and networks that are then able to offer free-of-charge access to their services and resources to researchers whose applications pass the competitive external review. One can distinguish (meta)data related to the service per se, and that related to any (meta)data produced as part of a scientific service during project implementation. For the former, the ISIDORe consortium invested significant effort into constituting a homogeneous catalogue of services with a harmonised metadata scheme for all of the more than 300 services offered. Furthermore, the scheme was chosen in consultation with the relevant experts involved in the BY-COVID project to ensure sustainability and interoperability. While most of the LS RIs have the possibility to incorporate their well established internal organisational service provision and access management procedures and operational guidelines into the project, for some of the participating networks in the consortium, the ISIDORe project represented an opportunity to formalise and harmonise the description of their services in a systematic manner. Facilitating the rationalisation of the entire catalogue, e.g. reducing duplications and having a common metadata scheme in place, will ensure that in the future, new services will necessarily be described using a common approach. The issues regarding (meta)data produced as part of a scientific service provided to an incoming research project are, however, more complicated. In part, this arises from the fact that the initial goal of many service providers has been to focus on their mission to provide open access to scientific services and resources to users for generating scientific data. The networks are, however, currently adopting targeted implementation of FAIR principles, and integration of domain-specific mandatory data management plans. This is also very much driven and facilitated by the competent RIs. In ISIDORe, for example, there is no consortium-wide user's charter detailing how users are expected, or required, to make their data FAIR, nor are instructions given to explain how sustainable data objects and data management components provided by EOSC/EOSC-Life can be used. Further, only a subset of participating RIs have a Data Management Plan (DMP) in place to help users make data collected during their project FAIR and bring it to open access data repositories in the respective scientific domain. This is at odds with the requirements specified for Horizon Europe, that beneficiaries should at least prepare a DMP, deposit data in a trusted repository and provide open access to it (‘as open as possible, as closed as necessary’), as well as provide information about any research output or any other tools and instruments needed to re-use or validate the data. A programme like ISIDORe partially provides a loop-hole regarding openness of data acquired using Horizon Europe funding. For some users, data deposition can be a challenging process. With their collective expertise, the RIs are in a unique position to help users fulfil the EC's requirements and provide substantial efforts to document their challenges and associated processes (preprint: David et al., 2023b). Future efforts will build on existing examples, such as the process to facilitate submission of sequence data to the European Nucleotide Archive. This has been proposed by ELIXIR (Harrison et al., 2021), with the support of BY-COVID, a FAIR EOSC-related project. Having such tools in place would then make adherence to open data rules much easier for users, so that this could then reasonably become a condition of service provision. This will, however, require broader awareness of the issues, as well as the provision of training and support, before and during the execution on research projects supported by ISIDORe, or other similar programmes. Working collectively on these issues within ISIDORe, in collaboration with BY-COVID, using outputs from EOSC-Life, should allow universal adoption of common and sustainable methods to allow RIs to facilitate data deposition by users in a form that is most amenable to future access by interested third parties. For ISIDORe partners, a real inclusive and efficient adoption of sustainable components for FAIR data sharing will depend on sustainable support from expert personnel and continuous training to leverage FAIR data sharing skills and literacy (David et al., 2020).

These strategies rely typically on a combination of income generated by the users’ fees, public funding at the European and national level (the latter often directed to the RIs’ national nodes and/or to support access to the RI services by national researchers), provision of services to third parties, such as companies or other research institutions. In certain cases, subscription fees are used to support central services i.e. the hub, but if the goal is to promote open access for all, this latter cannot be a preferred model. In recent years, there have been numerous examples of online scientific services and tools being offered free of charge to the scientific community, and once they have become widely adopted, being placed behind a paywall. This is the least desirable outcome if the goal is to ensure equitable global access to scientific data.

**References**

European Commission, Directorate‐General for Research and Innovation, Schwardmann U, Fenner M, Hellström M, Koers H, L'Hours H, Matthews B, Ritz R, Valle M et al (2021) PID architecture for the EOSC – Report from the EOSC Executive Board Working Group (WG) Architecture PID Task Force (TF), Publications Office

Harrison PW, Lopez R, Rahman N, Allen SG, Aslam R, Buso N, Cummins C, Fathy Y, Felix E, Glont M et al (2021) The COVID‐19 Data Portal: accelerating SARS‐CoV‐2 and COVID‐19 research through rapid open access data sharing. *Nucleic Acids Res* 49: W619–W623

Knapp J, Zeratsky J, Kowitz B (2016) *Sprint: how to solve big problems and test new ideas in just five days*, p 288. New York, NY: Simon & Schuster

Kolodkin AN, Sharma RP, Colangelo AM, Ignatenko A, Martorana F, Jennen D, Briedé JJ, Brady N, Barberis M, Mondeel TDGA et al (2020) ROS networks: designs, ageing, Parkinson's disease and precision therapies. *NPJ Syst Biol Appl* 6: 34

McGovern NY (2018b) Forward together. *Res Libr Issues* 296: 62–67

Nurnberger A (2018) The radical collaboration of RDA and what it means for developing institutional data management services. *Res Libr Issues*296: 23–32

Scott K (2017) *Radical candor: how to get what you want by saying what you mean*, 1st edn. London, UK: Pan Macmillan

Scott K (2019) *Radical Candor: How to get what you want by saying what you mean*, 2nd edn. London, UK: Macmillan

Tamm JW, Luyet RJ (2010) *Radical collaboration: five essential skills to overcome defensiveness and build successful relationships*, 2nd edn. New York, NY: HarperCollins

1. <https://sph.umich.edu/cehr/pdf/Paper_Sprint_Manual.pdf> [↑](#footnote-ref-1)
2. ELIXIR, BBMRI-ERIC, EATRIS-ERIC, ECRIN-ERIC, EMBRC, EMPHASIS, ERINHA, EU-OPENSCREEN, Euro-BioImaging, INFRAFRONTIER, Instruct-ERIC, ISBE, MIRRI [↑](#footnote-ref-2)
3. <https://www.emphasisproject.eu/> [↑](#footnote-ref-3)
4. <https://www.mirri.org/> [↑](#footnote-ref-4)
5. <https://www.eosc-life.eu/industrycall/> [↑](#footnote-ref-5)
6. <https://fragalysis.diamond.ac.uk/viewer/react/landing/> [↑](#footnote-ref-6)
7. <https://open-phytoliths.github.io/FAIR-phytoliths/> [↑](#footnote-ref-7)
8. <https://www.infrafrontier.eu/> [↑](#footnote-ref-8)
9. <https://www.embrc.eu/emo-bon> [↑](#footnote-ref-9)
10. <https://docs.mgnify.org/src/docs/analysis.html> [↑](#footnote-ref-10)
11. <https://oneworldanalytics.com/abspermits/> [↑](#footnote-ref-11)
12. <https://www.cbd.int/doc/c/e6d3/cd1d/daf663719a03902a9b116c34/cop-15-l-25-en.pdf> [↑](#footnote-ref-12)
13. <https://fairsharing.org/community_champions> [↑](#footnote-ref-13)
14. <https://fairsharing.org/educational> [↑](#footnote-ref-14)
